# A Sensitive and Specific Neural Signature for Picture-Induced Negative Affect

**DOI:** 10.1371/journal.pbio.1002180

**Published:** 2015-06-22

**Authors:** Luke J. Chang, Peter J. Gianaros, Stephen B. Manuck, Anjali Krishnan, Tor D. Wager

**Affiliations:** 1 Department of Psychology & Neuroscience, University of Colorado, Boulder, Colorado, United States of America; 2 Department of Psychology, University of Pittsburgh, Pittsburgh, Pennsylvania, United States of America; California Institute of Technology, UNITED STATES

## Abstract

Neuroimaging has identified many correlates of emotion but has not yet yielded brain representations predictive of the intensity of emotional experiences in individuals. We used machine learning to identify a sensitive and specific signature of emotional responses to aversive images. This signature predicted the intensity of negative emotion in individual participants in cross validation (*n* =121) and test (*n* = 61) samples (high–low emotion = 93.5% accuracy). It was unresponsive to physical pain (emotion–pain = 92% discriminative accuracy), demonstrating that it is not a representation of generalized arousal or salience. The signature was comprised of mesoscale patterns spanning multiple cortical and subcortical systems, with no single system necessary or sufficient for predicting experience. Furthermore, it was not reducible to activity in traditional “emotion-related” regions (e.g., amygdala, insula) or resting-state networks (e.g., “salience,” “default mode”). Overall, this work identifies differentiable neural components of negative emotion and pain, providing a basis for new, brain-based taxonomies of affective processes.

## Abstract 2

By using images to induce negative emotions in human participants, this study uses neuroimaging to develop and validate a distributed brain signature of emotion that can predict the magnitude and type of negative affective experience in new individuals.

## Author Summary

Emotions are an important aspect of human experience and behavior; yet, we do not have a clear understanding of how they are processed in the brain. We have identified a neural signature of negative emotion—a neural activation pattern distributed across the brain that accurately predicts how negative a person will feel after viewing an aversive image. This pattern encompasses multiple brain subnetworks in the cortex and subcortex. This neural activation pattern dramatically outperforms other brain indicators of emotion based on activation in individual regions (e.g., amygdala, insula, and anterior cingulate) as well as networks of regions (e.g., limbic and “salience” networks). In addition, no single subnetwork is necessary or sufficient for accurately determining the intensity and type of affective response. Finally, this pattern appears to be specific to picture-induced negative affect, as it did not respond to at least one other aversive experience: painful heat. Together, these results provide a neurophysiological marker for feelings induced by a widely used probe of negative affect and suggest that brain imaging has the potential to accurately uncover how someone is feeling based purely on measures of brain activity.

## Introduction

Emotions are a class of psychological states comprised of physiological responses, expressive behavior, and subjective experiences that are central to our daily lives and to multiple forms of psychopathology [1] and chronic medical diseases [2]. Emotional information organizes physiological, cognitive, and motor systems into adaptive [3], organism-wide responses to events and situations relevant for survival and well-being [4–6]. These responses allow us to pursue resources and avoid harm [7], translate cognitive goals into motivated behavior [8], and navigate the social world [9,10]. Conversely, emotional dysregulation is at the heart of many brain- and body-related disorders (e.g., mood, anxiety, personality, cardiovascular, and substance use disorders) and likely cuts across traditional diagnostic boundaries [11]. Thus, understanding the neurobiological mechanisms that generate and mitigate negative emotional experience is paramount to understanding both human flourishing and dysfunction.

The importance of understanding the “emotional brain” has motivated hundreds of neuroimaging studies in healthy humans [12,13] and those suffering from psychopathology [14–16]. The promise of these studies for basic research is that they will permit a brain-based taxonomy of emotional processes, avoiding the sole reliance on psychological categories [17,18], while the hope for clinical development is to provide transdiagnostic markers for psychopathology that can identify functional brain dysregulation [19] and physical health risk [2,20], predict treatment response [21,22], and guide new, brain-based treatments [23,24].

In spite of this promise, fundamental requirements must be met before neuroimaging findings can be considered brain representations of emotion that are useful for translational purposes [25]. Previous work has identified many brain correlates of emotional versus nonemotional stimuli [12] and physiological responses [26,27] but has yet to uncover brain signatures diagnostic of an individual’s emotional experience. For example, the amygdala, dorsal anterior cingulate (dACC), anterior insula (aINS), and other regions reliably respond to aversive stimuli [28], and functional alterations in these regions are considered prominent features of anxiety disorders [14,29]. However, activation in these regions does not imply an emotional experience. Amygdala activation can occur in the absence of emotional experience [30] and does not appear to be involved in all aversive experiences [31]. In addition, the dACC and aINS are among the most frequently activated regions in the brain across all types of emotional and nonemotional states [28] and have recently been conceptualized as network “hubs” that may be integrating cognitive, emotional, and motivational information [32,33].

One factor that contributes to this limitation is that the vast majority of studies focus on comparing types of stimuli [12], e.g., “negative” versus “neutral” images, rather than finer grained differences in reported experience [34]. While these emotion-related comparisons are assumed to reflect “affective processing,” confounds with attention, salience, and other processes may render many findings superfluous to emotional experience.

Thus, there is a pressing need for neural signatures that are optimized to predict emotional experiences and functional outcomes. These indicators should: (1) specify a precise set of brain voxels that can be tested in new individuals and prospectively applied to new samples and (2) be sensitive and specific to a class of affective experiences (e.g., negative emotion and not other states such as attention or arousal) [35].

Machine learning provides a new toolbox of algorithms suited for developing sensitive and specific signatures of psychological processes [36–39], particularly when those signatures involve measures across multiple neural systems, as is likely to be the case with emotional experience [12,18,40]. Standard neuroimaging methods generally preclude estimation and optimization of the strength of the brain experience correspondence [28,41–43], but cross validated machine learning analyses can identify whether brain effects are of sufficient magnitude (e.g., sensitive enough) and specific enough to have translational utility. These techniques have recently shown great promise in identifying patterns that discriminate among types of affective experiences from brain [35,44–46] and physiology [47], discriminating patient from control groups [19,48], and predicting treatment response [49].

Here, we use machine learning in a large sample *(n* = 183) to identify the brain systems that predict the intensity of negative affective experiences elicited by viewing images from the International Affective Picture System (IAPS) [50], which is among the most robust methods of eliciting brief affective experiences (d = 0.81) [51]. In spite of the widespread use of IAPS images in basic and clinical research (e.g., it is the primary affective task in the human connectome project [52]), the brain mechanisms that underlie the genesis of the negative experiences they evoke have not been clearly identified. In addition, it is unclear (a) whether it is possible to identify a pattern that strongly predicts emotional experience prospectively in out-of-sample individuals, (b) which brain systems are involved (cortical, subcortical, or both), and (c) whether brain activity that tracks negative affect is specific for negative affect, or whether it codes for “salience,” arousal, or more general features of stimulus processing. Answers to all of these questions are critical for continued progress in both basic affective and clinical sciences.

We address each of these questions by developing a multivariate pattern that predicts negative emotion and assess its sensitivity and specificity relative to pain—another type of arousing, salient, negative experience. Finally, to examine the distributed versus localized nature of the signature, we examined the subsystems necessary and sufficient for accurately predicting negative emotional experience.

## Results

### The PINES Signature

**Fig 1:**
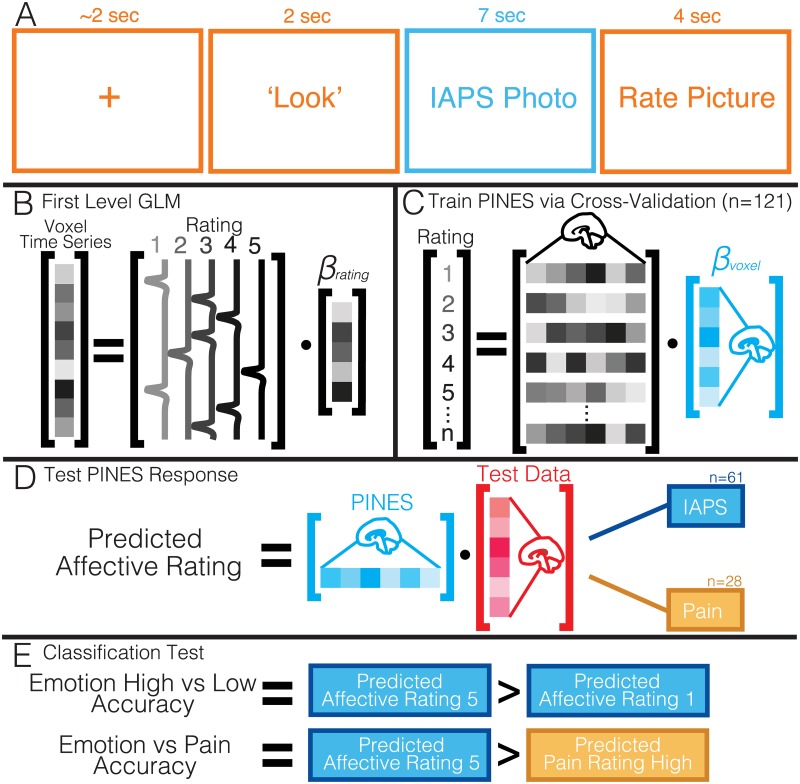
Experimental paradigm and analysis overview. Panel A depicts the sequence of events for a given trial. Participants view an initial fixation cross and then are instructed to look at the picture (compared to reappraise). Participants then see a photo and are asked to rate how negative they feel on a likert scale of 1–5. Panel B illustrates the temporal data reduction for each rating level using voxel-wise univariate analysis and an assumed hemodynamic response function. Panel C: these voxels are then treated as features and trained to predict ratings using LASSO-PCR with leave-one-subject-out cross validation. Subject’s data for each rating is concatenated across participants. Panel D: this multivoxel weight map pattern can be tested on new data using matrix multiplication to produce a scalar affective rating prediction. Panel E: we calculated two different types of classification accuracy: (a) the ability to discriminate between high (rating = 5) and low (rating = 1) affective ratings and (b) the ability to discriminate between high affective and high pain data.

We used Least Absolute Shrinkage and Selection Operator and Principle Components Regression (LASSO-PCR) [35,53] to identify a distributed Picture Induced Negative Emotion Signature (PINES) that monotonically increased with increasing affective ratings in leave-one-subject-out cross validated analyses *(n* = 121). To apply the model to data from individual test subjects in both cross validation (*n* = 121) and separate hold-out test datasets (*n* = 61), we calculated the pattern response—the dot product of the PINES weight map and the test image—for individual subjects’ activation maps for each of 5 levels of reported negative emotion (see Fig 1). The resulting continuous values reflect the predicted intensity of negative emotion for a given activation map. We used these values to classify which of two conditions elicited a stronger negative emotion for an individual (a “forced-choice” test) [35], providing accuracy estimates (Fig 1E). We also used similar classification tests, described below, to evaluate the sensitivity and specificity of PINES responses to negative emotion versus pain. We focus primarily on results for the test sample, as it was completely independent of all model-training procedures and provides the strongest evidence for generalizability [54].

**Fig 2:**
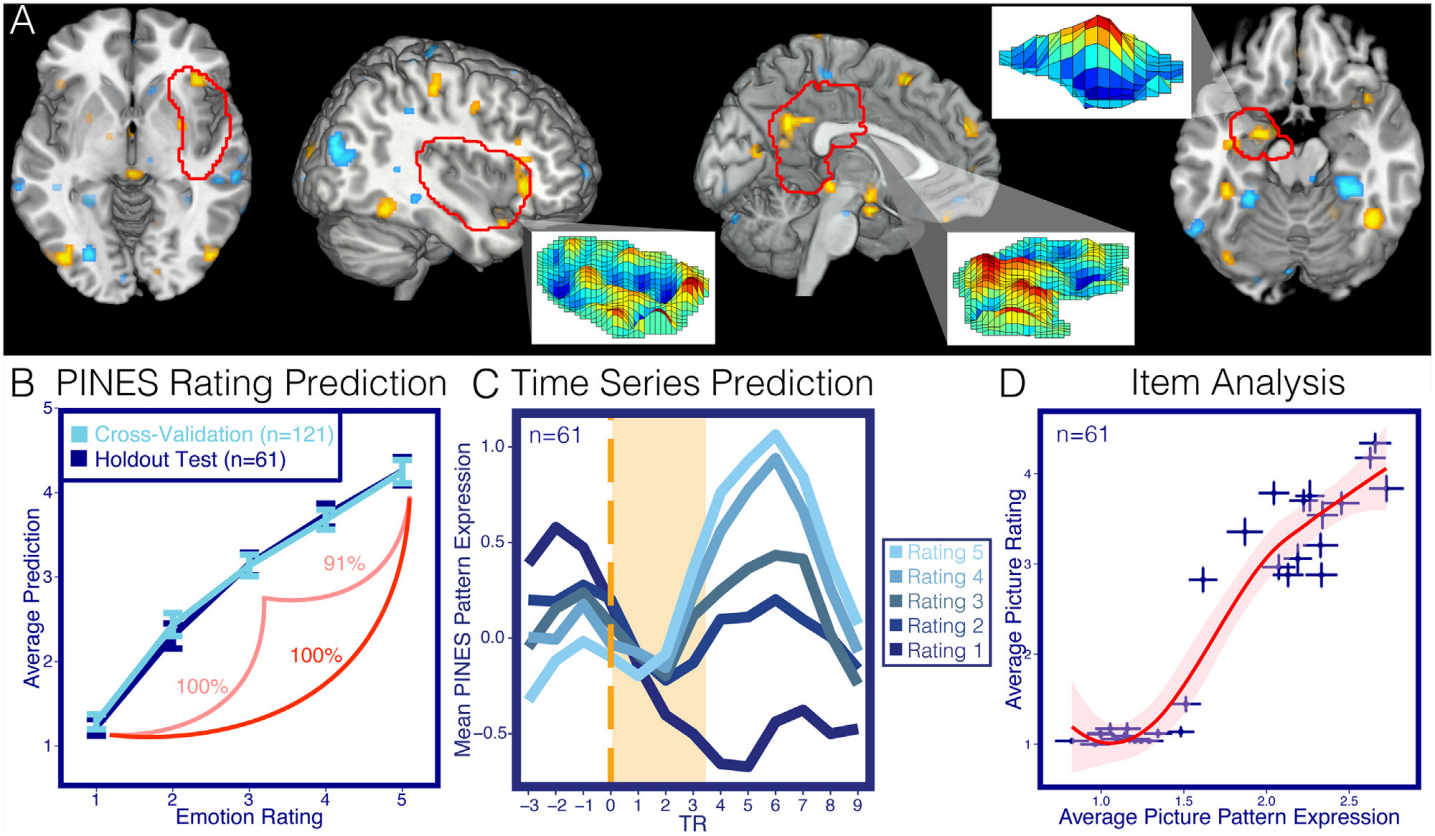
PINES. Panel A depicts the PINES pattern thresholded using a 5,000 sample bootstrap procedure at *p* < 0.001 uncorrected. Blowout sections show the spatial topography of the pattern in the left amygdala, right insula, and posterior cingulate cortex. Panel B shows the predicted affective rating compared to the actual ratings for the cross validated participants (*n* = 121) and the separate holdout test data set (*n* = 61). Accuracies reflect forced-choice comparisons between high and low and high, medium, and low ratings. Panel C depicts an average peristimulus plot of the PINES response to the holdout test dataset (*n* = 61). This reflects the average PINES response at every repetition time (TR) in the timeseries separated by the rating. Panel D illustrates an item analysis which shows the average PINES response to each photo by the average ratings to the photos in the separate test dataset (*n* = 61). Error bars reflect ±1 standard error.

The PINES accurately predicted ratings of negative emotional experience in both cross validation and hold-out test datasets (Fig 2). For individual participants in the cross validation sample, the average root mean squared error (RMSE) was 1.23 ± 0.06 (standard error; SE) rating units, and the average within-subject correlation between predicted and actual ratings was r = 0.85 ± 0.02). Accuracy was comparable in the test sample (RMSE = 0.99 ± 0.07, r = 0.92 ± 0.01). The PINES accurately classified highly aversive (rating 5) versus nonaversive (rating 1) pictures with 100% forced-choice accuracy in both cross validation and test samples (Fig 2B). Classification accuracy was also high in both the highly aversive range (rating of 5 versus 3: forced-choice = 91%; test sample) and the moderately aversive range (rating of 3 versus 1: 100%; test sample) (See S1 Table). We also assessed single-interval classification based on a single image rather than a relative comparison (Table 1), which were only slightly less accurate (Table 1). Comparisons with Support Vector Regression (SVR), another popular algorithm, indicate that these results appear to be robust to the choice of algorithm and, to a large extent, the amount of data used in the training procedure (see S1 Methods).

**Table 1:** Patten sensitivity and specificity.

	Map	Emotion 5 versus 1 (SE)	Pain High versus Low (SE)	Emotion versus Pain (SE)^☨^	Emotion Correlation (SE)	Pain Correlation (SE)
Pattern						
	PINES	93.6 (2.6%)^+^	60.7 (8%)	93.2 (2.9%)^+^	0.92 (0.01)	0.64 (0.11)
	Neurologic Pain Signature (NPS)	27.7 (5%)^+^ *	82.1 (5.6%)^+^	10.7 (3.6%)^+^ *	-0.35 (0.06)	0.91 (0.04)
Average Region of Interest (ROI)						
	Amygdala	55.3 (6%)*	64.3 (8%)	50.5 (5.8%)*	0.31 (0.07)	0.62 (0.09)
	Anterior Cingulate (ACC)	55.3 (5.6%)*	75 (6.7%)^+^	50.5 (5.8%)*	0.26 (0.07)	0.9 (0.02)
	Insula	55.3 (6%)*	78.6 (6.2%)^+^	45.6 (5.7%)*	0.32 (0.07)	0.92 (0.02)
Network						
	Visual	50 (6.5%)*	57.1 (8%)	78.6 (4.7%)^+^ *	-0.01 (0.08)	0.22 (0.13)
	Somatomotor	36.2 (6.2%)^+^ *	71.4 (7.1%)^+^	28.1 (5.2%)^+^ *	-0.38 (0.06)	0.78 (0.09)
	Dorsal Attention	57.4 (6.4%)*	71.4 (6.2%)^+^	61.2 (5.6%)*	0.34 (0.07)	0.57 (0.12)
	Ventral Attention (Salience)	51.1 (6%)*	71.4 (6.2%)^+^	13.5 (3.9%)^+^ *	0.14 (0.07)	0.56 (0.13)
	Limbic	57.4 (6%)*	35.7 (8%)	53.4 (5.8%)*	0.28 (0.06)	-0.5 (0.13)
	Frontoparietal	51.1 (5.8%)*	60.7 (7.6%)	42.7 (5.7%)*	0.29 (0.07)	0.34 (0.13)
	Default	63.8 (5.4%)^+^ *	57.1 (7.6%)	70.8 (5.3%)^+^ *	0.34 (0.06)	-0.03 (0.15)

All balanced accuracies reported in this table result from single-interval classification on the test dataset *(n* = 47; see S1 Table for forced-choice test). Analyses involving Level 5 and/or Level 1 comparisons exclude participants that did not rate any stimuli with that label. Accuracy values reflect the ability to discriminate the conditions compared, but are signed, so that values >50% indicate the proportion of participants for which high intensity was classified as greater than low intensity, for high vs. low analyses, or emotion was greater than pain, for Emotion vs. Pain analyses. Values < 50% indicate the proportion of participants for which low intensity was classified as greater than high intensity or pain was classified as greater than emotion. For example, the 10.7% emotion classification of the NPS in the Emotion vs. Pain analysis should be interpreted as a 89.3% hit rate in discriminating pain from emotion. Correlations reflect Pearson correlations between participant’s pattern responses to levels of affective intensity and self-reported ratings averaged across participants.

^☨^Please note that this column does not reflect accuracy but rather percent classified as emotion.

^+^Indicates that accuracy is significantly different from chance (50%), using a two-tailed dependent binomial test.

*Indicates accuracy significantly different from PINES performance using a two-sample two-tailed z-test for proportions (only tested on Emotion 5 versus 1 and Emotion versus Pain columns).

The PINES pattern included reliable predictive weights across a number of cortical and subcortical regions (Fig 2A). Positive weights (greater activity predicts more negative emotion) were found in many regions typically associated with negative emotion [12,40], including the amygdala, periaqueductal gray (PAG), aINS, dorsomedial prefrontal cortex (dmPFC), ventral occipital cortex, presupplementary motor area (preSMA), ventromedial temporal lobe (mTL), and posterior cingulate cortex (PCC). Negative weights were found in the bilateral parahippocampal gyrus, right superior temporal gyrus, left temporal parietal junction (TPJ), right caudate, and occipital and somatomotor cortices. These regions likely comprise multiple functional systems, as we describe in more detail below. Though the PINES comprises nonzero predictive weights across the brain (see S1 Fig), supplementary analyses indicated that a sparse pattern thresholded at *p* < .001, as shown in Fig 2 (1.6% of in-brain voxels), was sufficient to predict emotional experience with comparable sensitivity to the full model (see S1 Methods and S5 Fig).

#### Moderation by demographic variables

An important issue for any biomarker is whether the relationship between predicted (i.e., PINES responses) and observed responses (i.e., negative emotion) is different for different subject populations. Here, all participants (*n* = 182) demonstrated a positive association between the magnitude of the PINES response and negative emotion. In addition, the slope of the relationship between the PINES response and test participants’ (*n* = 61) ratings was not moderated by demographic variables, including age (F(1,56) = 0.37, *p* = 0.54), sex (F(1,56) = 0.80, *p* = 0.38), ethnicity (Caucasian (86% of sample) versus African American (13%); F(1,56) = 0.29, *p* = 0.59), or IQ (F(1,56) = 0.96, *p* = 0.33).

#### Chronometry of the PINES response

To characterize the time course of PINES responses during and after picture viewing, we applied the PINES pattern to the entire timeseries in the test dataset (*n* = 61) and examined responses at each time point following stimulus onset (Fig 2C). Monotonic increases with negative emotional experiences began approximately 4 sec following picture onset and peaked at approximately 6 sec following picture offset, validating the adequacy of the hemodynamic model used here and verifying that the PINES response is linked to responses during picture viewing.

#### Item analysis

IAPS images vary in multiple ways, including the visual and social content of the images. To test whether the PINES pattern predicted negative emotion across the various images included in this study, we performed an item analysis to test how strongly the PINES expression correlated with ratings across individual pictures in the test dataset. We found a strong linear relationship between the average ratings and pattern response for each image r = .95, t(28) = 15.66, *p* < 0.001, even within only the negative pictures r = .67, t(13) = 3.22, *p* = 0.006 (Fig 2D). This finding suggests that the PINES response reflects emotional experience across the heterogeneous visual characteristics (e.g., color, luminance, spatial frequency) and content in the pictures. One potential confound is that most of the negative images depicted a social scene, while most of the neutral images were nonsocial. S7 Fig shows an item analysis highlighting the PINES response to counterexamples of each condition; the results suggest that the signature is not driven by the degree of sociality. In addition, the average PINES response across these images also strongly correlated with normative ratings of multiple discrete emotions from an independent study [55] (sadness, r = 0.92; anger, r = 0.94; disgust, r = 0.94; and fear, r = 0.88). This suggests that the PINES may be tracking a general negative emotional response rather than a specific emotional state, consistent with previous factor analyses of emotional elicitation using the IAPS [55]. However, as the normative emotion ratings are highly intercorrelated in this set of images, further studies are needed to examine the mapping between PINES responses and emotional content in more detail.

#### Within-subject prediction

**Fig 3:**
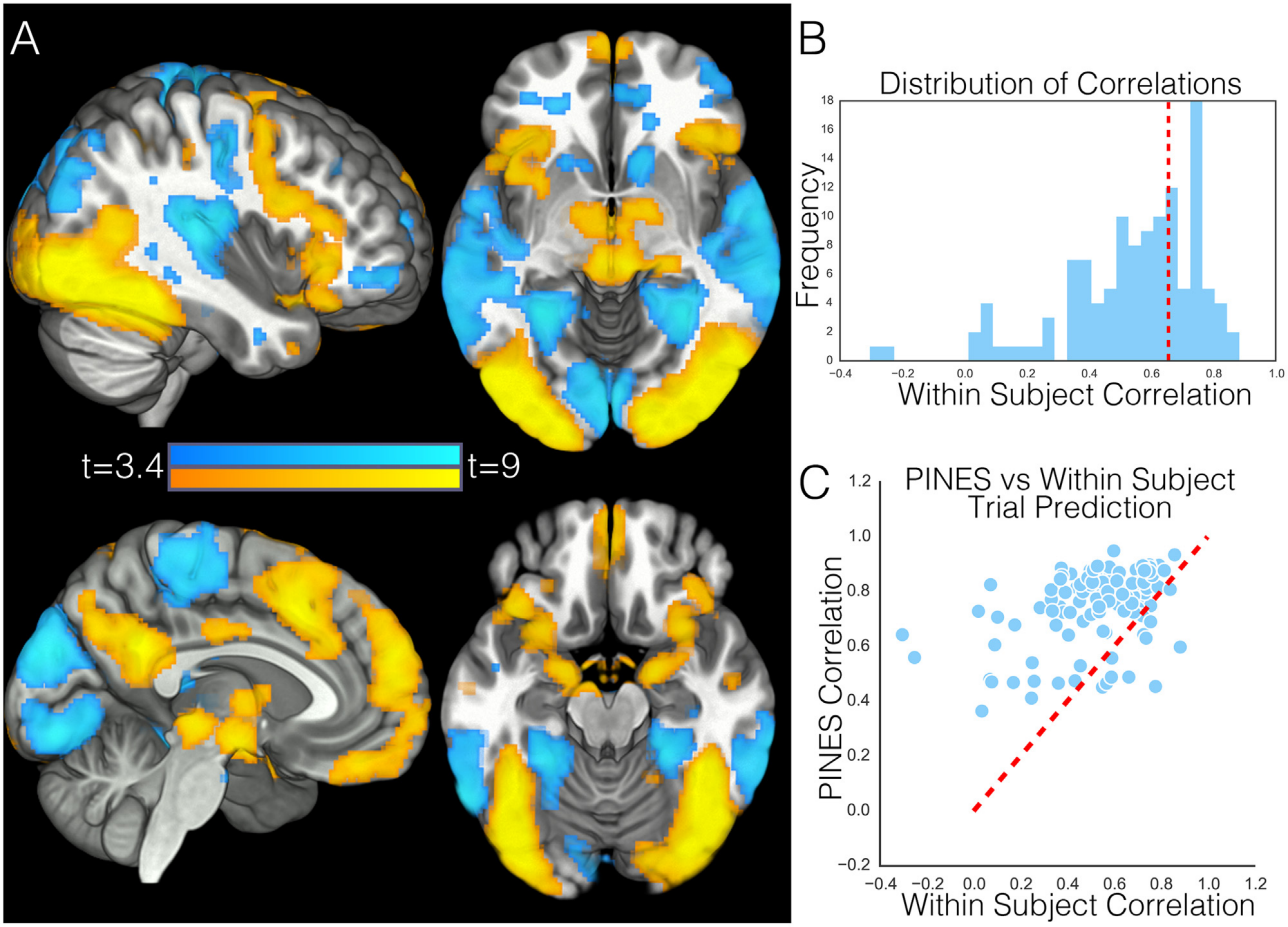
Within participant emotion prediction. This figure depicts results from our within-participant analysis, in which the PINES was retrained separately for each participant to predict ratings to individual photos. Panel A shows the voxels in the weight map that are consistently different from zero across participants using a one sample *t* test thresholded at *p* < 0.001 uncorrected. Panel B shows a histogram of standardized emotion predictions (correlation) for each participant. The dotted red line reflects the average cross validated PINES correlation for predicting each photo’s rating. Panel C depicts how well each participant’s ratings were predicted by the PINES (y-axis) versus an idiographically trained, cross-validated map using their individual brain data (x-axis). Each point on the graph reflects one participant. The dotted red line reflects the identity line. Any data point above the identity line indicates that the participant was better fit by the PINES than their own weight map.

In addition, it is important to assess the degree of individual variability in the spatial pattern of the PINES. It is possible that some brain regions important for affect may be highly variable across participants in both interparticipant spatial registration and functional topography. Therefore, in this analysis, we looked at the performance of patterns trained on individual participant data. Overall, the individualized predictive maps were able to predict affect ratings on individual trials (mean cross validated r = 0.54 ± 0.02). Interestingly, the cross validated PINES performed significantly better than the within-subject patterns (mean trial-by-trial r = 0.66 ±0.01), t(120) = 6.28, *p* < 0.001 (Fig 3C). The relative high accuracy of the PINES can be attributed to larger amounts of between-participant than within-participant trial data. The spatial topography of the average within-participant predictive map was similar to the PINES (spatial correlation r = .37), though the peaks of the most predictive regions were more spatially diffuse (see Fig 3A, S1 Fig). No individual participant’s weight map was more spatially similar to the PINES than the group mean (average r = 0.11 ± 0.01), which suggests that the individualized maps were much noisier than the PINES. The tradeoff between using the group (PINES) to regularize predictions compared to the individual alone reflects a classic bias and variance tradeoff fundamental throughout statistics. Introducing some bias towards the group can reduce variance in estimation, improving estimates and predictions. This is the general principle underlying empirical Bayes estimation, which is widely used throughout the statistical sciences.

#### Dependence on visual processing

Though the PINES includes weights in ventral occipital and temporal regions, which may be related to emotional experience or aspects of high-level visual processing correlated with emotion, accurate predictions do not depend on these regions. Accuracy for the test sample was maintained even when the PINES was retrained excluding the entire occipital lobe (forced-choice rating 5 versus 1 = 100%, RMSE = 0.96 ± 0.06, r = 0.89 ± 0.01; S2 Fig).

### Pattern Specificity

Affect systems may be organized by valence so that a brain signature for negative affect may be found across stimulus modalities and contexts, or in a modality-specific manner, such that there is not one “negative affect system” but many. Testing these hypotheses requires comparing multiple types of negative affect across modalities. Here, we assessed the generalizability and specificity of the PINES response across IAPS pictures and somatic pain, which is a negative, arousing experience arising from a different modality.

**Fig 4:**
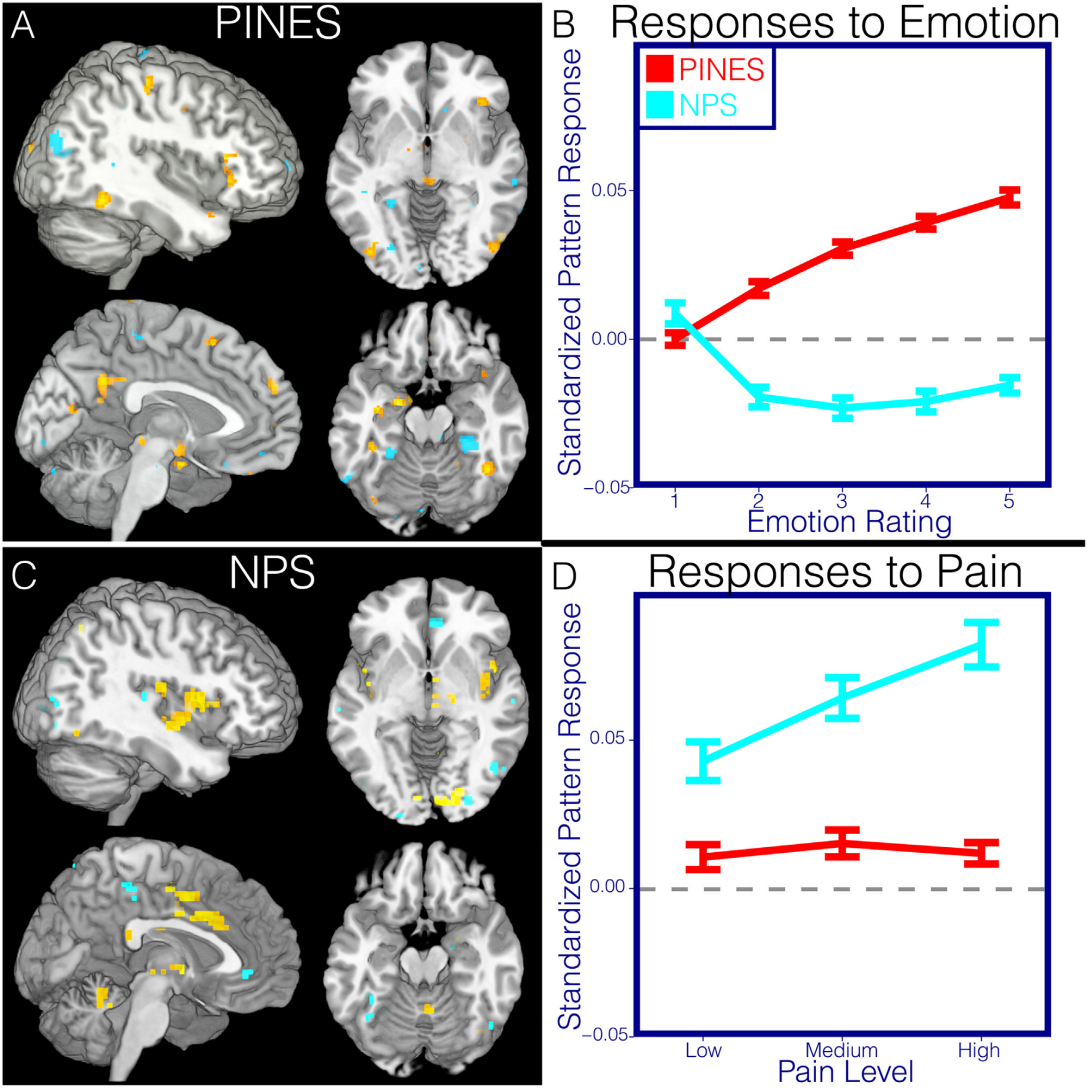
Affective and pain responses to PINES and NPS. This figure illustrates differences in the spatial topography in the thresholded PINES and NPS patterns and their predictions in independent emotion (*n* = 61) and pain (*n* = 28) test data. Panel A depicts the PINES thresholded at *p* < 0.001 uncorrected (see Fig 2). Panel B depicts the average standardized PINES and NPS pattern responses at each level of emotion calculated using a spatial correlation. Error bars reflect ±1 standard error. Panel C depicts the NPS thresholded at false discovery rate (FDR) q < 0.05 whole-brain corrected. Panel D depicts the average standardized PINES and NPS pattern responses at each pain level calculated using a spatial correlation. Error bars reflect ±1 standard error.

**Fig 5:**
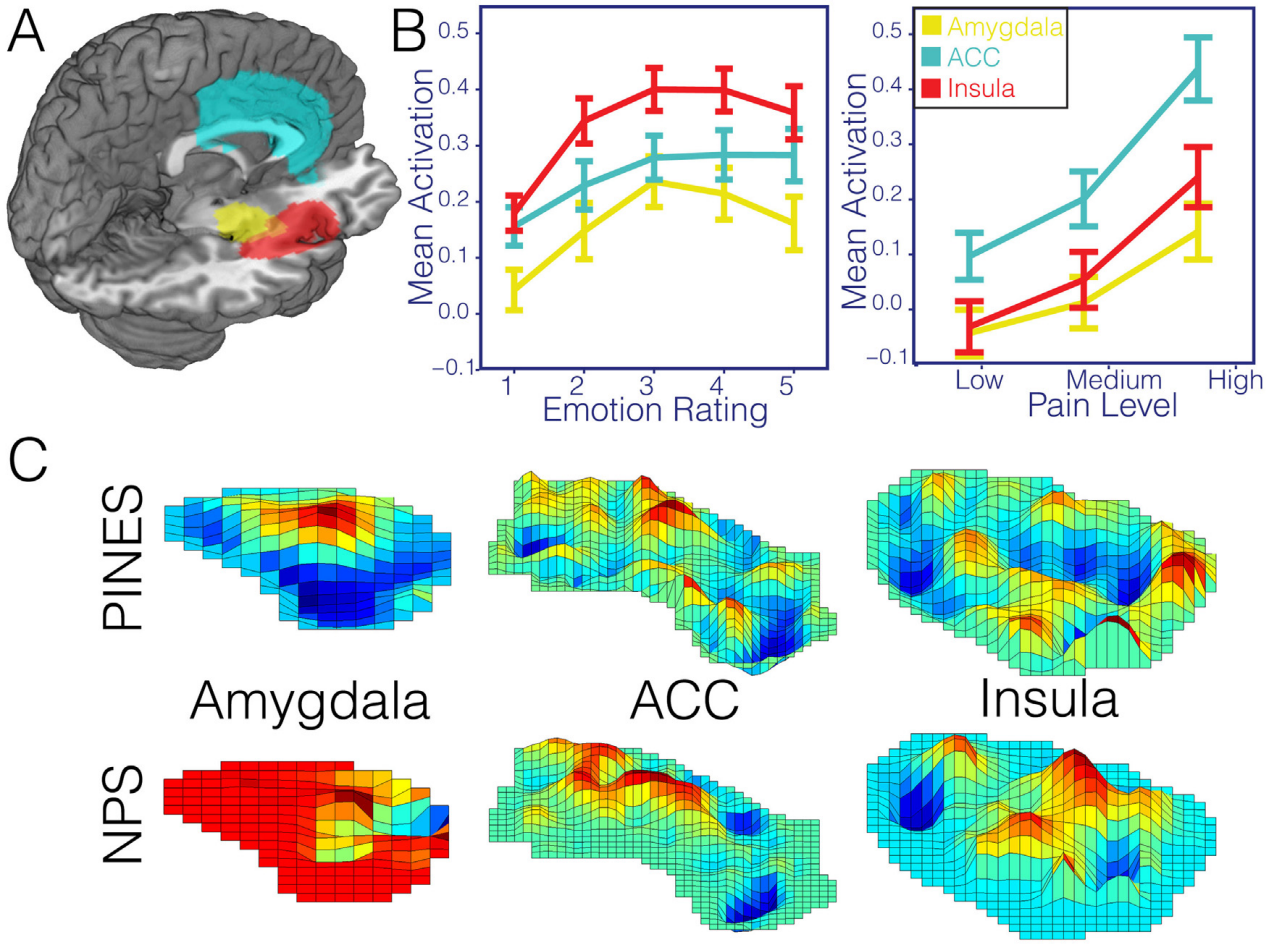
Region of interest analysis. Panel A illustrates the spatial distribution of the three anatomical ROIs used in all analyses (amygdala = yellow, insula = red, ACC = cyan). Panel B depicts the average activation within each ROI across participants for each level of emotion and pain in the emotion hold out (*n* = 61) and pain test datasets (*n* = 28). Error bars reflect ±1 standard error. Panel C illustrates the spatial topography of the PINES and NPS patterns within each of these anatomical ROIs. While these plots show one region, correlations reported in the text reflect bilateral patterns.

We employed two types of analyses to examine the PINES specificity. First, we compared the spatial topography of the PINES to another pattern map, the Neurologic Pain Signature (NPS), which shows high sensitivity and specificity to somatic pain across multiple studies [35]. The PINES and NPS maps were almost completely uncorrelated (robust ranked spatial correlation, $\hat{\rho }$ = −0.01; Fig 4). Several regions showed positive weights in both maps, including the anterior cingulate (ACC), insula, and amygdala. As shown in Fig 5C, however, the weight patterns within these regions were also uncorrelated (bilateral ACC, $\hat{\rho }$ = 0.04, insula, $\hat{\rho }$ = −0.05), though weights in the amygdala were modestly correlated ($\hat{\rho }$ = 0.21).

Second, we assessed the specificity of the pattern responses in the test IAPS (*n* = 61) and thermal pain (*n* = 28) [56] datasets. The PINES accurately predicted negative affect in the IAPS dataset (*n* = 61) but showed no response to increasing pain intensity in the pain dataset (Fig 4). Conversely, the NPS responded robustly to increasing pain but showed no response to increasing negative affect in the IAPS dataset. To further assess sensitivity and specificity, we examined how well responses in each pattern could discriminate (a) high pain versus high negative affect, (b) high versus low pain, and (c) high versus low negative affect (Table 1). Because this involves comparing responses from two separate, imbalanced test sets (*n* = 61 versus *n* = 28), the analyses described below employ single interval classification, in which individual images are tested for suprathreshold responses independently (as compared to relative within-subject differences in forced-choice classification). The threshold was determined by finding the point that minimized signal detection response bias (see Methods for details), and we report balanced emotion classification accuracy (chance = 50%), sensitivity, and specificity (See S2 Table for equivalent forced-choice analyses).

#### Pain versus emotion

The PINES responded more strongly to emotion than pain and accurately discriminated between the two (93.2 ± 2.9% [SE] accuracy, 93.6% sensitivity, and 92.9% specificity); conversely, the NPS responded more strongly to pain and accurately discriminated pain from emotion (89.3 ± 3.6% accuracy, 89.4% sensitivity, and 89.3% specificity). Thus, both patterns discriminated between the two modalities.

#### High versus low intensity in each modality

The NPS successfully classified high versus low pain (82.1 ± 5.1% accuracy, 82.1% sensitivity, and 82.1% specificity). It also significantly, though less accurately, classified high versus low emotion (Rating 5 versus 1; 70.4 ± 4.4%, 70.2% sensitivity, and 70.5% specificity), but NPS responses were actually strongest for low-intensity emotion. Examining responses across emotion levels revealed that this was caused by deactivation of the NPS for all levels of emotion >1, resulting in nonmonotonic responses. In contrast, the PINES successfully classified high versus low emotion ratings (93.5 ± 2.4% accuracy, 93.6% sensitivity, and 93.4% specificity) but was at chance in discriminating high versus low pain (60.7 ± 6.5% accuracy, 60.7%, sensitivity, 60.7% specificity).

Together, these analyses suggest that the PINES is specific to negative emotion, at least as compared with pain, and that both the PINES and NPS capture distinct aversive states. Importantly, we are only assessing specificity to one type of construct (e.g., pain) among many possible options. Future work must build on these initial novel observations to examine relationships across many types of positive and negative affect and stimulus modalities to provide a comprehensive picture of the organization of negative affect systems.

### PINES Outperforms Prediction Based on ROIs and Resting-State Networks

Another question is whether the precise pattern of activity specified in the PINES uniquely captures negative affect, or whether regions and networks previously used in the literature are sufficient. In order to fully appreciate the sensitivity and specificity of the PINES, it is necessary to compare it to the standard univariate approach, which typically examines average activation within ROIs compared to baseline activity. In this analysis, we examined the average response to emotion and pain stimuli within anatomical ROIs and canonical networks defined in large-scale resting-state studies [57].

#### PINES outperforms ROIs

We tested three a priori ROIs that play prominent roles in negative emotion: ACC, insula, and amygdala (see Table 1, Fig 5A). All ROIs showed linear increases across levels of emotion (Fig 5B; S1 Methods), but the effects were not strong enough to predict emotion ratings. For the amygdala, prediction—outcome correlations were positive (test dataset RMSE = 3.04 ± 0.05; r = 0.31 ± 0.07), but high versus low classification accuracy was at chance (55.3 ± 6%, sensitivity = 55.3%, specificity = 55.3%; Table 1). Comparable effects were found in the ACC and insula. For the ACC: RMSE = 2.96 ± 0.05, r = 0.32 ± 0.07, accuracy = 55.3 ± 5.6%, sensitivity = 55.3%, specificity = 55.3%. For the insula: RMSE = 2.88 ± 0.05, r = 0.37 ± 0.07, accuracy = 55.3 ± 6%, sensitivity = 55.3%, specificity = 55.3%. In addition, the amygdala and insula showed a nonmonotonic response function across levels of emotion (Fig 5), as evidenced by significant linear and quadratic effects in both regions (see S1 Methods). Together, these results indicate that averaged activity in these ROIs is not sufficient to predict emotional experience. See S1 Methods for a whole-brain searchlight analysis that shows local regions predictive of emotion and also local patterns that cross predict pain experiences.

#### PINES outperforms network maps

Many types of brain representations may be encoded in distributed functional networks, and there is a growing consensus that functional connectivity in a small set of canonical networks may capture some representations important for cognition and emotion [57–62]. In this analysis, we compared the PINES to predictive information in a popular seven-network whole-brain parcellation based on resting-state activity in 1,000 participants [57], treating each of the network masks as a pattern (see Methods). While “somatomotor” (accuracy = 63.8 ± 6.2%) and “default” (accuracy = 63.8 ± 5.4%) networks discriminated between high and low levels of emotion above chance, none of the networks performed nearly as well as the PINES (see Table 1 and S6 Fig for all results), including the “ventral attention network,” (also frequently referred to as the “salience” network) [57,63], which performed at chance in discriminating high versus low negative emotional experience (51.1 ± 6%).

Activity in four networks successfully discriminated between responses to emotion and pain (Table 1). The “visual” and “default mode” networks were more strongly activated by high levels of emotion than high levels of pain (78.6 ± 4.7% and 70.8 ± 5.3%), while the ventral attention and somatomotor networks responded more strongly to pain than emotion (86.5 ± 3.9% and 71.9 ± 5.2%, respectively). These results suggest that the brain patterns that define commonly used resting-state networks can identify the sensory modality of aversive stimulation with moderate accuracy, but they are not sufficient for predicting the intensity of emotional experience.

### The PINES Is Composed of Distinct Subnetworks

Defining a brain pattern sensitive and specific to a type of negative emotion is a critical first step towards developing meaningful models of brain representations of emotion. Here, the development of the PINES affords the opportunity to characterize the basis of this pattern representation within and across brain networks. Constructionist theories of emotion [12,18] predict that negative affect is created by interactions among discrete subnetworks that span multiple brain systems, whereas more traditional modular views predict that one system may be sufficient. We tested whether the PINES might be composed of multiple distinct subnetworks and whether responses in multiple subnetworks are necessary for predicting emotional responses. If so, the negative affect captured by the PINES might be considered a truly multisystem distributed process.

**Fig 6:**
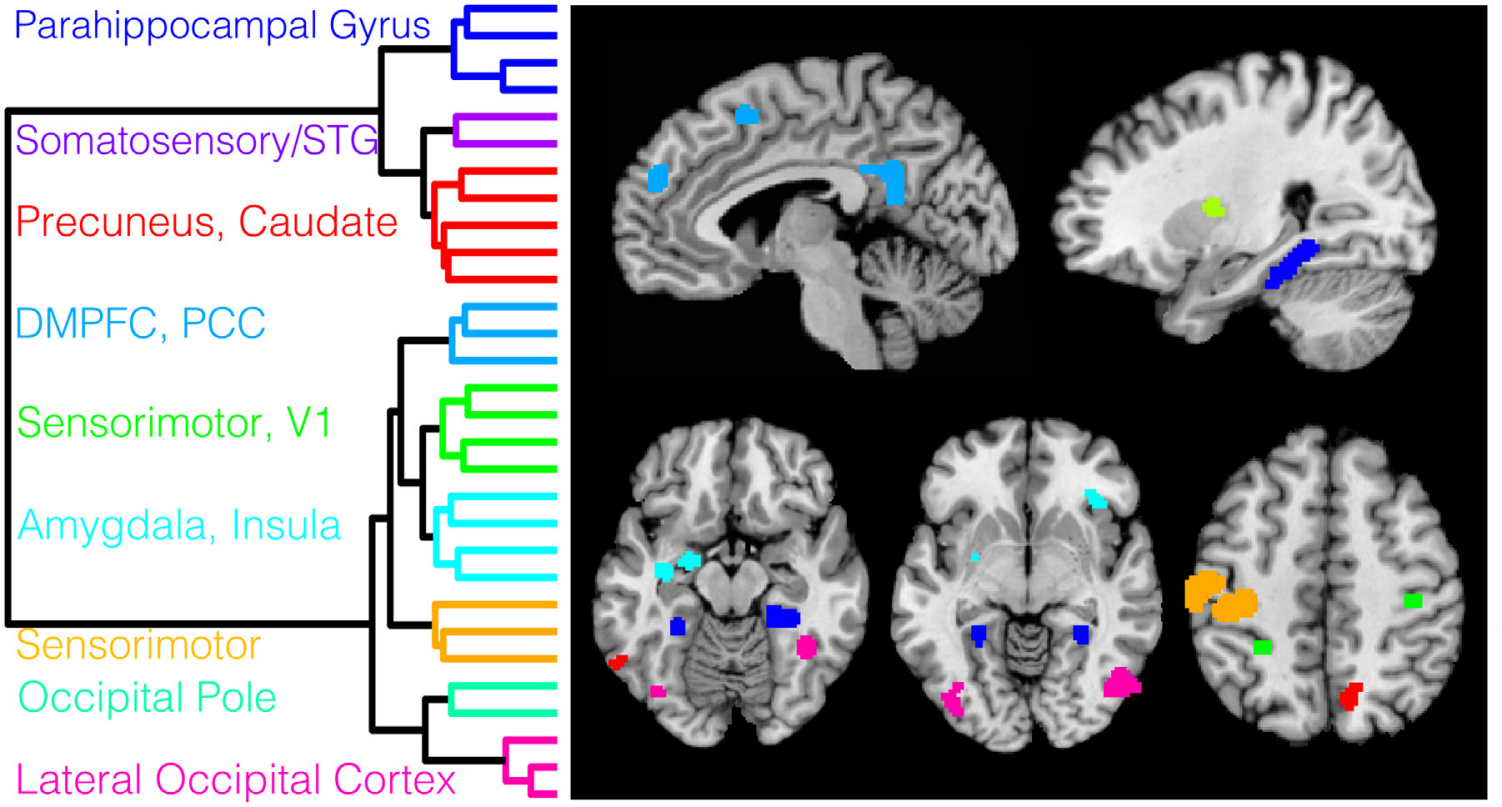
PINES clustering based on shared patterns of connectivity. This figure depicts the results of the hierarchical clustering analysis of the functional connectivity of the largest regions from the *p* < 0.001 thresholded PINES pattern. Clusters were defined by performing hierarchical agglomerative clustering with ward linkage on the trial-by-trial local pattern responses for each region using Euclidean distance. Data were ranked and normalized within each participant and then aggregated by concatenating all 61 subjects’ trial x region data matrices. Panel A depicts the dendrogram separated by each functional network. Panel B depicts the spatial distribution of the networks. Colors correspond to the dendrogram labels.

For this analysis, we calculated pattern responses within each of the largest regions in the PINES (*p* < .001, k = 10 voxels; see S1 Methods) for every individual trial within each participant and used a robust clustering algorithm to group the PINES regions into separate networks based on similar patterns of trial-by-trial covariation (see Methods). The best solution contained nine separate clusters, which provides a descriptive characterization of the subnetworks that comprise the PINES (Fig 6, S3 Table) that is broadly consistent with constructionist accounts of emotion [12] and previous meta-analyses of emotion-related networks [17]. These subnetworks included (a) two networks encompassing different parts of the visual cortex (e.g., lateral occipital cortex [LOC] and occipital pole) consistent with the visual modality of the stimuli, (b) a left amygdala-right aINS-right putamen network, which has been implicated in multiple forms of arousal and salience, (c) a network that includes bilateral posterior parahippocampi and the precuneus, which are broadly involved in memory and other forms of contextual processing, and (d) a network that includes parts of the dmPFC and PCC that are likely involved in social cognition but are distinct from more executive processes [64,65]. An additional network that includes the right somatosensory cortex and contralateral cerebellum may be involved in preparing for the rating action but may also play a more fundamental role in the emotion generation process [66].

#### “Virtual lesion” analysis

Because the PINES is a statistical model of a distributed neural representation of emotion, it is possible to evaluate how well subsets of the brain model can predict emotional experiences. An interesting question is whether each subsystem is either necessary or sufficient to predict the emotional experience. Thus, in a “virtual lesion” analysis, we tested (a) how well each network cluster could discriminate between high versus low ratings and between pain versus emotion and (b) how much the predictive accuracy was reduced by removing each network from the predictive map. As expected by the substantial discrepancies in the task modalities (e.g., visual pictures versus thermal pain and motor response in the IAPS task), several networks individually performed well at discriminating between the emotion and pain including the visual LOC (85.4 ± 4.1%), occipital pole (85.4 ± 4.1%), and sensorimotor and cerebellar networks (93.2 ± 2.9%). These networks were also able to discriminate between levels of emotion significantly above chance, but importantly, all were significantly outperformed by the PINES (see Table 2). Removing individual networks resulted in slight decreases in high versus low emotion classification accuracy but had a negligible difference on discriminating between pain and emotion. Classification accuracy remained above 80% for high versus low emotion and above 90% for emotion versus pain after removing each single network, and none of these were significantly different from the PINES. These analyses indicate that no specific subsystem was either necessary or sufficient for predicting negative affect, supporting the multisystem view.

**Table 2:** Single-cluster and “virtual lesion” analysis.

	Map	nVoxels	Emotion 5 versus 1 (SE)	Pain H versus L (SE)	Emotion versus Pain (SE)	Emotion Correlation (SE)	Pain Correlation (SE)
Pattern							
	PINES	328796	93.5 (2.4%)	60.7 (6.5%)	93.2 (2.9%)	0.92 (0.01)	0.64 (0.11)
	PINES (*p* < .001)	5303	91.5 (3%)^+^	67.9 (7.6%)^+^	97.2 (1.9%)^+^	0.89 (0.01)	0.51 (0.13)
Single Cluster							
	Visual (LOC)	981	83 (4.3%)^+^ *	64.3 (7.1%)^+^	85.4 (4.1%)^+^	0.73 (0.03)	0.56 (0.12)
	Somatosensory and superior temporal gyrus (STG)	308	59.6 (5.8%)*	32.1 (7.1%)^+^	61.2 (5.6%)*	0.12 (0.07)	-0.66 (0.11)
	Sensorimotor and V1	335	57.4 (6.2%)*	67.9 (7.6%)^+^	57.3 (5.7%)*	0.23 (0.07)	0.8 (0.07)
	DMPFC and PCC	318	70.2 (5.4%)^+^ *	60.7 (7.6%)	70.8 (5.3%)^+^ *	0.47 (0.06)	0.61 (0.1)
	Sensorimotor and Cerebellum	1227	78.7 (4.5%)^+^ *	60.7 (7.6%)	93.2 (2.9%)^+^	0.72 (0.04)	0.39 (0.14)
	Parahippocampal Gyrus	1025	51.1 (6.4%)*	39.3 (7.1%)	39.9 (5.7%)*	-0.05 (0.07)	-0.43 (0.13)
	Occipital Pole	118	55.3 (6.7%)*	53.6 (8%)	85.4 (4.1%)^+^	0.29 (0.08)	0.22 (0.14)
	Precuneus and Caudate	537	48.9 (6.2%)*	28.6 (7.1%)^+^	53.4 (5.8%)*	-0.15 (0.07)	-0.82 (0.06)
	Amygdala and Insula	454	59.6 (6%)*	75 (6.7%)^+^	54.4 (5.7%)*	0.39 (0.06)	0.76 (0.08)
Virtual Lesion-Cluster Removed	Visual (LOC)	4322	85.1 (4%)^+^	46.4 (8.4%)	96.1 (2.3%)^+^	0.72 (0.05)	-0.17 (0.13)
	Somatosensory and STG	4995	91.5 (3%)^+^	64.3 (8%)	93.2 (2.9%)^+^	0.87 (0.01)	0.67 (0.11)
	Sensorimotor and V1	4968	95.7 (2.1%)^+^	50 (8%)	97.2 (1.9%)^+^	0.9 (0.01)	0.08 (0.15)
	DMPFC and PCC	4985	89.4 (3.4%)^+^	57.1 (8.7%)	97.2 (1.9%)^+^	0.9 (0.01)	0.37 (0.14)
	Sensorimotor and Cerebellum	4076	91.5 (3%)^+^	60.7 (8.4%)	96.1 (2.3%)^+^	0.84 (0.02)	0.56 (0.11)
	Parahippocampal Gyrus	4278	85.1 (4%)^+^	67.9 (7.1%)^+^	96.1 (2.3%)^+^	0.83 (0.02)	0.62 (0.11)
	Occipital Pole	5185	93.6 (2.6%)^+^	64.3 (7.6%)	97.2 (1.9%)^+^	0.89 (0.01)	0.46 (0.14)
	Precuneus and Caudate	4766	89.4(3.4%)^+^	66.1(7.8%)^+^	96.1(2.3%)^+^	0.85(0.02)	0.76(0.07)
	Amygdala and Insula	4849	91.5(3%)^+^	57.1(8.4%)	97.2(1.9%)^+^	0.9(0.01)	0.25(0.15)

All balanced accuracies reported in this table result from single interval classification on the test sample (*n* = 47; see S2 Table for forced-choice test). Analyses involving Level 5 and/or Level 1 comparisons exclude participants that did not rate any stimuli with that label. Accuracy values reflect the ability to discriminate the conditions compared, but are signed so that values >50% indicate the proportion of participants for which high intensity was classified as greater than low intensity for high vs. low analyses, or emotion was greater than pain for Emotion vs. Pain analyses. Values < 50% indicate the proportion of participants for which low intensity was classified as greater than high intensity or pain was classified as greater than emotion. For example, the 10.7% emotion classification of the NPS in the Emotion vs. Pain analysis should be interpreted as a 89.3% hit rate in discriminating pain from emotion. Correlations reflect Pearson correlations between participant’s pattern responses to levels of affective intensity and self-reported ratings averaged across participants.

^+^Indicates that accuracy is significantly different from chance (50%) using a two-tailed binomial test.

*Indicates accuracy is significantly different from PINES performance using a two-sample, two-tailed z-test for proportions (only tested on Emotion 5 versus 1 and Emotion versus Pain columns).

## Discussion

For neuroimaging to be useful in translational applications (e.g., psychiatry, neurology, etc.), sensitive and specific brain signatures must be developed that can be applied to individual people to yield information about their emotional experiences, neuropathology, or treatment prognosis [25]. Thus far, the neuroscience of emotion has yielded many important results but no such indicators for emotional experiences. Signatures that are sensitive and specific for particular affective processes are presumably much closer to brain representations of emotional experience, which can then be interrogated to better understand the mechanisms and typology of emotion at the neurophysiological level.

The goals of the present study were to: (a) develop a brain signature capable of reliably and accurately predicting the intensity of negative emotional responses to evocative images, (b) characterize the signature’s performance in generalizing across individual participants and images, (c) examine its specificity related to another negative and arousing affective experience (pain), and (d) explore the structure of the subnetworks necessary and sufficient to predict negative emotional experience.

We used cross validated machine learning analyses to identify a distributed pattern of activity predictive of emotional experiences, which we term PINES. The PINES fulfills the basic criteria for a brain signature of negative affect. It accurately predicted monotonic increases in negative affect ratings in 93.5% of individual test participants (*n* = 61; single interval). In forced-choice tests, it correctly identified which of two sets of images was rated as more negative in 90%–100% of individuals, as long as the images differed by two or more subjective rating points (on a five point scale). This demonstrates sensitivity to negative affect across the full range of the measurement scale.

PINES responses were also surprisingly specific to negative emotion. The PINES did not respond to increased levels of physical pain, another type of arousing, aversive, salient experience. Conversely, the NPS [35]—a signature previously found to be sensitive and specific to physical pain—responded strongly to physical pain but not to increasing levels of picture-induced emotional intensity. This double dissociation implies that neither pattern is driven by general arousal, salience, or negative affect. Though the PINES and NPS independently tracked the intensity of negative affect elicited in visual and somatosensory modalities, respectively, we do not believe they are dissociable based simply on differences in sensory processing for two reasons: (1) the PINES was just as accurate in predicting negative emotion without the occipital lobe, and when subnetworks associated with modality-specific processes were removed (e.g., visual, somatosensory, etc.) and (2) the local PINES and NPS patterns within traditional “affect” regions, such as the ACC and insula, were uncorrelated. This is consistent with our previous work demonstrating that pain is distinct from other emotional processes based on distributed spatial topography both across brain regions [28] and within local regions [35,67].

Further analyses explored the nature of emotion-predictive brain representations. The PINES was comprised of multiple separable subnetworks. Each network independently contributed to the prediction of participants’ negative emotion ratings controlling for other brain regions, and no single network was necessary or sufficient for predicting emotional experience. This pattern of results suggests that the PINES is a distributed pattern that encompasses a number of functional systems, and that multiple systems are required to capture negative affective experience.

### Implications for Theory and Measurement of Emotion

These results have theoretical implications for the neurobiology of emotion in terms of both the diversity of processes underlying affective experiences and how they are represented in the brain. Emotions are often defined as a composite of multiple intrinsically inter-related processes (e.g., autonomic arousal, expressive behavior, action tendencies, interoception, and conscious experiences). Theories differ widely on how these processes combine to give rise to emotional experience [1], but most major theories suggest that cognitive, sensory, motor, motivational, and interoceptive processes are critical ingredients of emotional experience. For example, appraisal theories view emotion as a dynamically unfolding process and emphasize the role of appraisals [8,68,69], embodied affect theories emphasize interoceptive and somatomotor representations [70], and constructionist theories view emotions as being constructed from all of these component processes [12,18].

In spite of this richness, since MacLean [71], theories of the emotional brain have treated emotion as a singular faculty that is localizable to a specific system. Often, this view has translated into “structure-centric” theories of emotional experience; e.g., the amygdala is critical for fear [72], the ACC for pain affect [73], and the insula for disgust [74]. In other cases, this view translates into circumscribed pathways or networks for “core affect” [17] and emotional awareness [75].

It remains unclear how far the structure-centric view can take us in understanding the brain bases of emotional experience. The regions most strongly identified with emotion are also intimately involved in a wide array of cognitive functions such as attention, error monitoring, associative learning, and executive control [33]. Recent connectivity [63] and functional diversity analyses [32] suggest that these regions are not solely processing affective signals but rather represent functional “hubs” for integrating many types of information.

As the limitations of the structure-centric view are increasingly widely recognized [12,33], researchers have moved towards the identification of intrinsically connected networks conserved both at rest and during active tasks [76]. These networks have been labeled with process-general names including the “salience network” [63], “default mode” network [77], and others, and a modern incarnation of the “emotional brain” theory suggests that the basis of emotional experience is encapsulated in one or a few of these networks such as the “limbic” network named after MacLean’s original formulation.

Our results corroborate the view that structure-centric—and even network-centric—models of emotion are limited and provide an alternative model for the brain representation of emotional experience. In this study, we targeted the conscious experience component, which is the defining feature of subjective distress and suffering. None of the anatomical regions identified in previous literature (e.g., amygdala, ACC, insula) predicted the intensity of emotional experience or discriminated emotion from pain in this study. This suggests that the effects identified in previous work using traditional statistical parametric mapping approaches are small and unlikely to serve as effective signatures of the type or magnitude of an emotional experience in an individual person.

Furthermore, activity in predefined networks was insufficient to capture negative emotion ratings, demonstrating that the pattern we identified using targeted machine-learning analysis is not reducible to these more process-general networks. The fact that networks and regions defined a priori, even from very large resting-state samples [57], were insufficient to capture emotional experience here has broad implications for the study of emotion and attempts to identify biomarkers for mental health disorders going forward [21,25,49].

Finally, our clustering analysis of the PINES map indicated that multiple, separable subnetworks distributed widely throughout the brain made independent contributions to predicting emotional experience. Importantly, no single subnetwork appeared to be necessary or sufficient in characterizing the emotional experience, as the accuracy in predicting the magnitude or type of experience did not significantly decrease when any given network was omitted. This pattern is consistent with both appraisal [68,69] and constructionist theories of emotion [12,78], which posit that emotional experiences result from interactions between core affect, sensory, memory, motor, and cognitive systems [40].

### Conclusions, Limitations, and Future Directions

Overall, these results provide an important step towards identifying emotion-related patterns that can serve as indicators for components of emotional experience. Such signatures can be used as intermediate phenotypes for genetic or risk-stratification studies, and they may provide objective neurobiological measures that can supplement self-report. The identification of intermediate brain-based phenotypes is critical, as self-reported emotion can be affected by many independent processes [8,18,68]—e.g., core experience, self-reflection, decision-making heuristics, and communicative intentions—which have different implications for understanding what exactly treatments that modulate emotion are measuring and which processes are affected by interventions.

We close with several key points and future directions. Importantly, the PINES is not necessarily a biomarker of negative emotion in general. We have demonstrated that it is a signature for the type of affect induced by aversive IAPS images, but its transferability to other emotional states (e.g., emotion induced by recall, rejection, positive emotion, or stress) remains to be tested. Such tests are a long-term program of future research that must span many studies and papers. We still know very little about the underlying structure of affect and which types of emotional responses can be cross predicted by the same brain markers. It is possible that the PINES captures some types of negative emotion and not others, and findings to this effect will help us move beyond the categories proscribed in our language to develop a more nuanced, brain-based view of affective processes [7,17].

In addition, testing the specificity and transfer of the PINES across many different kinds of affect is a key to developing more robust and specific markers. The PINES can undoubtedly be improved. For example, with further development and testing, it may be differentiated into markers for more specific types of emotional experiences (e.g., emotion categories like fear, disgust, etc. or canonical affect-inducing appraisals). In addition to types of affect, the PINES can be tested for responses across patient groups (e.g., schizophrenia, depression, or anxiety) and treatments thought to affect emotion (e.g., self-regulation, drug treatment, psychotherapy, etc.). This study provides a foundation and a benchmark for such future developments.

## Methods

All participants provided written informed consent, and experimental procedures were approved by the Institutional Review Board of the University of Pittsburgh for the IAPS study and the University of Colorado, Boulder for the pain study.

### IAPS Data Set

#### Participants

One hundred eighty three participants (mean age = 42.77 y, SD = 7.3 y; female = 52%; Caucasian = 87%) were recruited from the greater Pittsburgh area to participate in this study. One participant was excluded due to missing data. Participants were recruited from a larger study on health and full details regarding recruitment procedures, the study sample, and testing procedures have been previously reported [79]. The findings reported in this paper have not been previously reported and do not overlap with those in published reports from this larger study.

#### Stimuli

Task stimuli consisted of 15 negative photographs and 15 neutral photographs selected from the IAPS [50]. Pictures were presented using the E-Prime stimulus presentation software (Psychology Software Tools, Sharpsburg, PA). A small mirror was attached to the head coil enabling the viewing of projected images onto a screen while in the scanner. Negative photographs (Pictures: 2053, 3051, 3102, 3120, 3350, 3500, 35550, 6831, 9040, 9050, 9252, 9300, 9400, 9810, and 9921) depicted bodily illness and injury (ten photographs), acts of aggression (two photographs), members of hate groups (one photograph), transportation accidents (one photograph), and human waste (one photograph). Neutral photographs (Pictures: 5720, 5800, 7000, 7006, 7010, 7040, 7060, 7090, 7100, 7130, 7150, 7217, 7490, 7500, 9210) depicted inanimate objects (ten photographs) or neutral scenes (five photographs).

#### Task

Participants completed a reappraisal task and were instructed to either (a) “look” and maintain their attention to the photos when they came on screen and allow their emotional reactions to occur naturally or (b) “decrease” and change the way they thought about the image to feel less negative (see [79] for full task and IAPS stimulus details). Each trial consisted of a 2 sec instructional cue, “look”, followed by the 7 sec presentation of either a negative or neutral image. After the stimulus presentation, participants were given a 4 sec opportunity to report their emotional state using a 5-point Likert scale (where 1 indicated feeling neutral and 5 indicated feeling strongly negative). Finally, there was a variable (jittered) 1–3 sec rest period before the next cue (Fig 1A). We emphasized to participants to base their ratings on how negative they felt at the end of the image-viewing period. Only “look” trials were included in analyses for this paper. Though we use participants’ ratings to train and test our brain model, not every participant reported every level of emotion. For the training sample (*n* = 121), 98% used a “1,” 88% used a “2,” 98% used a “3,” 88% used a “4,” and 80% used a “5” in their response. For the test sample (*n* = 61), 100% used a “1,” 89% used a “2,” 93% used a “3,” 95% used a “4,” and 77% used a “5” in their response. The lower frequency of participants making a rating of “5” resulted in smaller sample sizes in “1” versus “5” rating accuracy tests.

#### Imaging data acquisition

Imaging data were acquired on a 3T Trio TIM whole-body scanner (Siemens, Erlangen, Germany) using a 12-channel, phased-array head coil. Each session included a structural scan for coregistration of the functional images (FOV = 200 × 200 mm, matrix = 256 ×256, TR = 3,000 ms, inversion time (TI) = 100 ms, TE = 11/101 ms, and FA = 150°, 36 slices, 3 mm thick, no gap) and a functional scan, which included 344 blood oxygen level dependent (BOLD) images (FOV = 200 × 200 mm, matrix = 64 × 64, TR = 2,000 ms, TE = 29 ms, flip angle (FA) = 90°, 34 3 mm slices with no gap).

#### Imaging preprocessing

fMRI data were preprocessed and analyzed using SPM8 (http://www.fil.ion.ucl.ac.uk/spm) and custom Matlab (MATLAB, The MathWorks, Inc., Natick, MA) code available from the authors’ website (http://canlab.colorado.edu). Images were first unwarped and realigned to the first image of the series using a six-parameter, rigid-body transformation. The realigned images were then coregistered to each participant’s T2-weighted structural image and normalized to the 152 MNI template using a 12-parameter nonlinear and affine transformation. Spatially normalized images were smoothed with a 6 mm full-width-at-half-maximum (FWHM) Gaussian kernel and filtered with a high pass filter (180 sec cutoff). When using whole-brain prediction, smoothing is thought to improve sensitivity to large-scale patterns [80], which can likely improve between subject predictions.

A univariate general linear model (GLM) was used to create images for the prediction analysis (Fig 1B). The model included one boxcar regressor indicating the rating period, to model any effects related to motor activity, and another modeling the mean picture viewing epoch. The model also include five separate boxcar regressors indicating the onset times for each IAPS picture, which allowed us to model brain activity in response to each picture separately for each rating level (e.g., [1–5]). All regressors were convolved with a double gamma HRF function, and an additional 24 covariate regressors modeled movement effects (6 realignment parameters demeaned, their 1st derivatives, and the squares of these 12 regressors).

### Pain Data Set

#### Participants

Thirty healthy, right-handed participants (Mean Age = 25.2 y, STD = 7.4 y, female = 40%) were recruited to participate in an fMRI study in which they received thermal pain stimulation (details on additional counterbalanced sessions is described in a separate manuscript [56]). Twenty-eight participants completed the thermal pain session. Participants with psychiatric, physiological, or pain disorders, and neurological conditions were excluded.

#### Pain calibration

All participants completed a pain calibration session to determine if they could tolerate the thermal stimulations that they would receive in the fMRI experiment. Thermal stimulation was applied on the volar surface of the left forearm and dorsal surface of the left foot using a TSA-II Neurosensory Analyzer (Medoc Ltd., Chapel Hill, NC) with a 16 mm Peltier thermode end plate. Three levels of thermal stimulation (pseudorandomly assigned to each participant)—low (44 or 45°C), medium (46 or 47°C), and high (48 or 49°C)—were applied to four different locations on both the upper limb (i.e., volar surface of the left forearm) and lower limb (i.e., dorsal surface of the left foot). Each stimulation lasted a total of 11 seconds with a 2 sec ramp-up, a 2 sec ramp-down, and 7 sec at the peak target temperature. The participants made responses on a Visual Analog Scale (VAS), which had anchors based on a labeled magnitude rating scale [81,82]. Participants first made a moment-by-moment rating where they used a pointer on the screen to move continuously along the rating scale and indicate the level of sensation they felt at each moment. They then made an overall rating at the end of each trial to indicate the maximum overall sensation they experienced in that trial. Participants who successfully completed the calibration procedure were then scheduled for the fMRI sessions.

#### fMRI session

Participants completed a separate scanning session for thermal pain that contained 11 runs and lasted about an hour. Each stimulation (i.e., 46, 47, and 48°C of heat) was preceded by a predictive cue (i.e., three levels of cues that corresponded to three levels of stimulation). Prior to being scanned, participants completed a short training in which they learned the levels of the three cues that were to be later presented in the scanner through an explicit learning task [83]. The first two fMRI runs consisted of a conditioning task where the participant learned the association between the cues they encountered in the prescan training and the level of stimulation for that session. During both the conditioning (two runs) and experimental runs (nine runs), participants received a cue—stimulus pair for each trial and were asked to make a rating on a visual analogue scale (same as the calibration session) about the sensation they felt after each trial. The participants rated the intensity of pain they felt during each trial. Each experimental run contained nine trials (81 total), which were counterbalanced for each participant using a Latin Square design. Experimental trials (i.e., postconditioning) began with a 2 sec cue followed by a systematic jitter separating the cue from stimulation (i.e., 5, 7, 11 sec). Participants then received stimulation for 11 sec followed by a jittered fixation (2, 6, or 14 s). The 11 sec trial duration for somatic pain included a 2 sec ramp-up, 2 sec ramp-down, and 7 sec at-target temperature. Finally, participants had 4 sec to make a rating of the sensation they experienced for the stimulation on a visual analogue scale using a trackball (responses were confirmed with a button click). There was an intertrial jittered fixation (1, 4, or 10 sec) that was counterbalanced across trials within a run so that all runs were of equal duration. Stimulus presentation and behavioral data acquisition were controlled using Matlab software.

#### Imaging acquisition

fMRI data were acquired on a Siemens Tim Trio 3T MRI scanner at the Intermountain Neuroimaging Consortium facility at the University of Colorado, Boulder. Structural images were acquired using high-resolution T1 spoiled gradient recall images (SPGR) for anatomical localization and warped to Montréal Neurological Institute or MNI space. Functional images were acquired with an echoplanar imaging sequence (TR = 1,300 ms, TE = 25 ms, field of view = 220 mm, 64 x6 4 matrix, 3.4 x 3.4 x 3.4 mm voxels, 26 interleaved slices with ascending acquisition, parallel imaging with an iPAT acceleration of 2).

#### Preprocessing

All images were preprocessed using SPM8 (Wellcome Trust Centre for Neuroimaging, London, UK) and custom Matlab functions. Mean structural T1-weighted images were computed for each participant from all imaging sessions. The mean structural images were then coregistered to the first functional image for each participant with an iterative procedure of automated registration using mutual information from the coregistration in SPM8 and manual adjustment of the automated algorithm’s starting point until the automated procedure provided satisfactory alignment, and were normalized to MNI space using SPM8, interpolated to 2 × 2 × 2 mm voxels.

Functional images were corrected for slice-acquisition-timing and motion using SPM8. They were then warped to SPM’s normative atlas using warping parameters estimated from coregistered, high-resolution structural images, interpolated to 2 × 2 × 2 mm voxels, and smoothed with an 8 mm FWHM Gaussian kernel.

Prior to preprocessing of functional images, global outlier time points (i.e., “spikes” in signal) were identified by computing both the mean and the standard deviation (across voxels) of values for each image for all slices. Mahalanobis distances for the matrix of slicewise mean and standard deviation values (concatenated) were computed for all functional volumes (time), and any values with a significant χ^2^ value (corrected for multiple comparisons) were considered outliers (less than 1% of images were outliers). The output of this procedure was later used as a covariate of noninterest in the first level models.

#### fMRI analysis

First-level GLM analyses were conducted in SPM8. The first six volumes of each run were discarded, and the nine experimental runs were concatenated for each participant (the first two conditioning runs were excluded). Boxcar regressors, convolved with the canonical hemodynamic response function, were constructed to model periods for the 2 sec cue presentation, the 5, 7, or 11 sec variable prestimulus fixation period, the 11 sec thermal stimulation, and the 4 sec rating periods. The fixation cross epoch was used as an implicit baseline. A high-pass filter of 224 sec was used for the somatic pain session, which was determined based on a first-level analysis on the two conditioning runs, in which the variance inflation factor was determined to be less than 5%. Contrasts of interest included the low, medium, and high stimulation period collapsed across cues (i.e., low, medium, and high) and body site (i.e., upper limb and lower limb).

### Analysis Methods

#### Machine learning

We used whole-brain multivariate machine learning pattern analysis [35,53] to find global patterns of brain activity that best predicted participants’ self-reported affective ratings (e.g., 1–5; Fig 1C). A machine-learning prediction algorithm simply refers to a function that uses a vector of features (independent variables) to predict the value of a continuous outcome variable (see [84] for an introduction to this approach in the context of fMRI analysis). Here, we used individual voxels of brain activity as features and used them to predict participants’ affective ratings of the pictures they viewed while undergoing fMRI. The LASSO-PCR algorithm [35,53] combines principal components regression with an L1 least squares regularization [85]. Each 3-D map of beta weights from the first level analysis was converted into a single vector and used to predict the affective rating value. Because there are considerably more voxels (*n* = 352,328) than subjects (*n* = 182), we first reduced the brain data into the same number of components as observations in our training dataset (*n* = 121) using a principal components analysis. Each component represents many different brain voxels that share a similar pattern. These components were then used to predict affective ratings using least squares regression with an L-1 regularization (LASSO). This regularization shrinks beta parameters to zero. Components with a nonzero beta were selected and then refit using OLS to ensure that they were not unduly affected by the shrinkage (see [85]). The betas were then back-projected into voxels in 3-D MNI space.

#### Cross validation

To minimize the possibility of our algorithm overfitting the data, we used a rigorous cross validation procedure [85,86]. The general principle of cross validation is to divide a dataset into two parts; one is used to “train” the classifier, and the other is used to “test” the accuracy of the classifier. This procedure minimizes overfitting and ensures that the predictive power of the algorithm generalizes beyond the training dataset. There are a variety of cross validation procedures, and here we used a very conservative stratified double cross validation approach to maximize the generalizability of our results [54]. We first divided the dataset into a training set (2/3 of sample, *n* = 121) and a final test dataset (1/3 of sample, *n* = 61) by stratifying the data along each participant’s average negative rating (this ensured that the average ratings were equal across groups). The training data were then subjected to a leave-one-subject-out cross validation. In this approach, N-1 participants’ beta images are trained to predict the corresponding affective rating, and then these weights are tested on the left out participant’s data. This process provides an efficient procedure to allow every data point to serve as both training and test data. The master holdout set, which was not used in the training cross validation procedure was then used to assess the final accuracy of the algorithm.

#### Determining predictive voxels

The cross validated PINES pattern consists of the weights of each voxel in predicting the affective rating plus the intercept. However, to determine the voxels that made the most reliable contributions to the classification, we performed a bootstrap test [87]. This involved taking 5,000 samples with replacement from the training dataset and repeating the prediction process with each bootstrap sample. We then converted this distribution into a z-value at each voxel and thresholded the map based on the corresponding *p*-value. We used multiple thresholds to highlight the regions that were most consistently predictive in the classification procedure (i.e., *p* < 0.01, 0.005, 0.001 uncorrected, and FDR *p* < 0.05 corrected). All predictions using the test data were performed with the full set of unthresholded weights, which included all nonzero voxels. We use *p* < 0.001 for display purposes as we found that this sparse map was able to predict responses almost as well as the full weight map (S2 Fig). However, we use the full weight map for all analyses reported in this manuscript.

#### Prediction

To test the performance of the PINES, we applied the pattern to novel test datasets to quantify the degree to which the pattern was expressed in other datasets. This approach is critical to assessing the convergent and discriminant validity of the PINES response [88]. The degree to which the PINES was expressed in other datasets, i.e., the PINES response (PR), was estimated for each test subject in each test condition by taking the dot product of the vectorized activation images (${\vec{\beta }}_{map}$) with the signature pattern (${\vec{W}}_{map}$), i.e., ($PR\;=\;{\vec{\beta }}_{map}\;\cdot \;{\vec{W}}_{map}$) yielding a single scalar value. Pattern response values were averaged across subjects in the test datasets. This method allows us to make a pointed prediction about the affective rating akin to regression. We also used spatial correlation to compare the relationship between the pattern and various maps. This method facilitates comparing relationships across maps with different numbers of voxels and intensities and is proportional to the dot product except scaled between −1–1. For binary maps (e.g., resting-state network parcellations [57]), we used point-biserial correlations, and for comparing specific regions of the weight maps, we used robust regression.

#### Within subject prediction

To examine the possibility of the prediction weights being influenced by poor anatomical registration, we ran an additional analysis in which we trained a separate model for each subject in the training sample (*n* = 121) and evaluated the consistency of each weight for every voxel in the brain across subjects using a one-sample *t* test. We used the LASSO-PCR algorithm with a 5-fold cross validation to predict participant ratings for individual IAPS pictures. We compared the performance of these individual weight maps to the cross-validated PINES map. This allowed us to test the performance of the PINES on an individual participant’s trial-level data without blurring the boundary between training and test data. Functional alignment techniques [89] are a promising solution to addressing issues concerning individual variability in anatomical registrations and should be considered in future work.

#### Evaluation

To evaluate the performance of the LASSO-PCR algorithm, we compared the model’s prediction with the actual rating by calculating the RMSE to illustrate overall prediction error and a Pearson correlation to indicate the effect size. Accuracy was determined using both forced-choice and single-interval classification methods from receiver operator characteristic curves (ROC). Forced-choice accuracy is “threshold free” in that it takes the maximum value of a relative comparison within a subject. It has an interesting property that the ROC curves are symmetrical, and sensitivity, specificity, and positive predictive value are equivalent to each other and to decision accuracy (i.e., the probability with which the more painful of the two conditions is selected). For single-interval classification, we used balanced accuracy [90] formalized as,

$$b=\;\frac{1}{2}\left(\frac{TP}{TP+FN}+\frac{TN}{FP+TN}\right)$$

where true positives (TP), true negatives (TN), false positives (FP), and false negatives (FN) are defined relative to the threshold that minimizes signal detection response bias [91]. We used a two-tailed dependent binomial z-test to assess the significance of single-interval classification accuracy, a two-tailed independent binomial test for forced-choice classification accuracy, and McNemar’s z-test for comparing two dependent proportions to compare the classification accuracy between the PINES and all other maps [92].

#### Clustering

We used hierarchical agglomerative clustering to find predictive regions that showed similar response profiles across trials. For this analysis, we extracted contiguous regions from the PINES that survived the *p* < .001 uncorrected threshold and contained a minimum of ten voxels. These regions provided the strongest contributions to the PINES. Region-specific pattern response values to each trial (*n* = 30) were rank ordered and normalized within-subject to (a) provide statistically robust connectivity estimates, as in nonmetric multidimensional scaling algorithms [93], and (b) reflect within-subject “beta-series” connectivity, which is both less susceptible to imaging artifacts than raw connectivity [94] and insensitive to individual differences in hemodynamic variables. Inter-region connectivity matrices were calculated aggregating across trials and subjects and subjected to hierarchical agglomerative clustering with Euclidean distance using the Ward minimum variance algorithm. Clusters were determined using an arbitrary threshold of 31% of the maximum distance, which resulted in nine distinct clusters.

## Supporting Information

S1 Data Trial level emotion and pain data. Contains information about each participant’s trial for the emotion datasets. Pain dataset is averaged within each pain level. File names correspond to nifti files located at http://neurovault.org. Pattern responses to PINES, the within-subject PINES, and each PINES cluster (e.g., C_*).
(CSV)

S2 Data Rating level emotion and pain data. Contains information about each participant’s average rating for the emotion and pain datasets. Contains average activation within ROIs, resting-state networks, PINES, and NPS pattern responses.
(CSV)

S1 Fig Weight maps. This figure depicts axial slice montages of different analytic techniques. PINES FDR: the PINES thresholded using a 5,000 sample bootstrap procedure at FDR q < 0.05 whole-brain corrected with a cluster extent k = 10. PINES-LASSO-PCR the full unthresholded PINES pattern trained with LASSO-PCR (*n* = 121). PINES-n182: the PINES weight map when it is trained with the full dataset (*n* = 182). PINES-SVR: the PINES when it is trained with the training data (*n* = 121) using support vector regression. PINES-within: the average weight map for the within-participant analysis, in which a separate pattern was trained for each participant to predict ratings to individual photos (*n* = 121). PINES-searchlight: standardized prediction values (i.e., correlations) for each voxel from a whole-brain searchlight analysis (*n* = 182).
(TIF)

S2 Fig PINES pattern response without occipital lobe. Panel A depicts the occipital mask excluded from the data prior to training the PINES. Panel B shows the predicted affective rating compared to the actual ratings for the cross validated participants (*n* = 121) and the separate holdout test dataset (*n* = 61).
(TIF)

S3 Fig Varying sample size prediction. This figure depicts the results for the analysis in which we ran 20 iterations predicting emotion ratings using varying sample sizes. Each iteration randomly sampled participants’ data without replacement. Panel A shows the average standardized prediction (correlation) for each sample size ranging from *n* = 2 through *n* = 121 in the test dataset (*n* = 61). Panel B shows the average single interval cross predicted accuracy for discriminating between high and low levels of pain. Panel C shows the average single interval accuracy discriminating between high levels of emotion and pain in the test dataset. Panel D shows the average spatial correlation of the weight map trained using each sample size with the PINES. Error bars in all panels reflect ±1 standard deviation.
(EPS)

S4 Fig Searchlight analysis. This figure depicts results from a whole-brain searchlight analysis in which we trained a searchlight (five voxel radius) to predict emotion rating using LASSO-PCR with the full dataset (*n* = 182) and 5-fold cross validation. Panel A shows the thresholded correlation values for each searchlight (*p* < 0.001, uncorrected). Panel B shows the distribution of the correlation values of all searchlights in the brain. The dotted line shows the cross validated PINES correlation (*n* = 121) for comparison.
(TIF)

S5 Fig Thresholded PINES pattern response. Panel A depicts the average forced-choice accuracy between high and low emotion ratings for the hold out test dataset (*n* = 61). The only threshold level that is significantly different from the PINES is the FDR q < 0.01 pattern. Panel B shows the average pattern correlation between each thresholded pattern for each emotion level. Panel C shows the average pattern correlation between each thresholded pattern for each level of pain. Error bars reflect ±1 standard error.
(TIF)

S6 Fig Network map pattern response. This figure examines how well resting-state networks perform using the same testing benchmarks as the PINES, NPS, and affective ROIs. The line plot depicts the pattern response of the network parcellation from Yeo et al., 2007 on emotion and pain test datasets using point-biserial spatial correlations. Panel A shows the average network predictions for each level of emotion, while panel B shows the average prediction for each level of pain. Error bars reflect ±1 standard error.
(TIF)

S7 Fig Item analysis with social and nonsocial images. This figure depicts the item analysis, in which the PINES pattern is tested on responses to individual photos in the test sample (*n* = 61). Error bars reflect ±1 standard error. The red item reflects the sole nonsocial negative image, while the cyan item reflects the sole social neutral image. This suggests that the PINES is not simply picking up on degree of socialness.
(EPS)

S8 Fig Cross prediction searchlight analysis. This figure depicts results from a whole-brain searchlight analysis in which we trained a searchlight (five voxel radius) to predict emotion rating using SVR with the training data set (*n* = 121) and 5-fold cross validation. We then applied each searchlight mask to the test pain data set (*n* = 28) to obtain a standardized pattern response, and calculated forced-choice accuracy within each participant to find searchlights that discriminated between high and low levels of pain. We show the accuracy results thresholded at *p* < 0.001 (note FDR q < 0.05 = *p* < 0.0015).
(TIF)

S1 Methods Contains supplemental analyses and results including: alternative PINES algorithms and analysis strategies, searchlight and cross-prediction analyses, thresholded PINES analyses, and anatomical ROI analyses. (DOCX)

S1 Table Pattern forced-choice classification. All balanced accuracies reported in this table result from forced-choice classification on the test dataset (*n* = 47). This analysis excludes participants that did not make a rating of either “1” or “5.” ^+^indicates that accuracy is significantly different from chance (50%) using a two-tailed independent samples binomial test. *indicates accuracy significantly different from PINES performance using a two-sample, two-tailed z-test for proportions.
(DOCX)

S2 Table Virtual lesion forced-choice classification. All balanced accuracies reported in this table result from forced-choice classification on the test dataset (*n* = 47). This analysis excludes participants that did not make a rating of either “1” or “5.” ^+^indicates that accuracy is significantly different from chance (50%) using a two-tailed independent samples binomial test. *indicates accuracy significantly different from PINES performance using a two-sample, two-tailed z-test for proportions.
(DOCX)

S3 Table Thresholded PINES clusters (*p* < 0.001, k = 10). (DOCX)

## Abbreviations

ACC: anterior cingulate
aINS: anterior insula
dACC: dorsal anterior cingulate
dmPFC: dorsomedial prefrontal cortex
FDR: false discovery rate
IAPS: International Affective Picture System
LASSO-PCR: Least Absolute Shrinkage and Selection Operator and Principle Components Regression
LOC: lateral occipital cortex
mTL: ventromedial temporal lobe
NPS: neurological pain signature
PAG: periaqueductal gray
PCC: posterior cingulate cortex
PINES: Picture Induced Negative Emotion Signature
preSMA: presupplementary motor area
RMSE: root mean squared error
ROI: region of interest
SE: standard error
STG: superior temporal gyrus
SVR: support vector regression
TPJ: temporary parietal junction
