# Supplementary figures and images for: A Sensitive and Specific Neural Signature for Picture-Induced Negative Affect
Source: PLoS Biol. 2015 Jun 22;13(6):e1002180. doi: 10.1371/journal.pbio.1002180 (PMC4476709; doi:10.1371/journal.pbio.1002180)

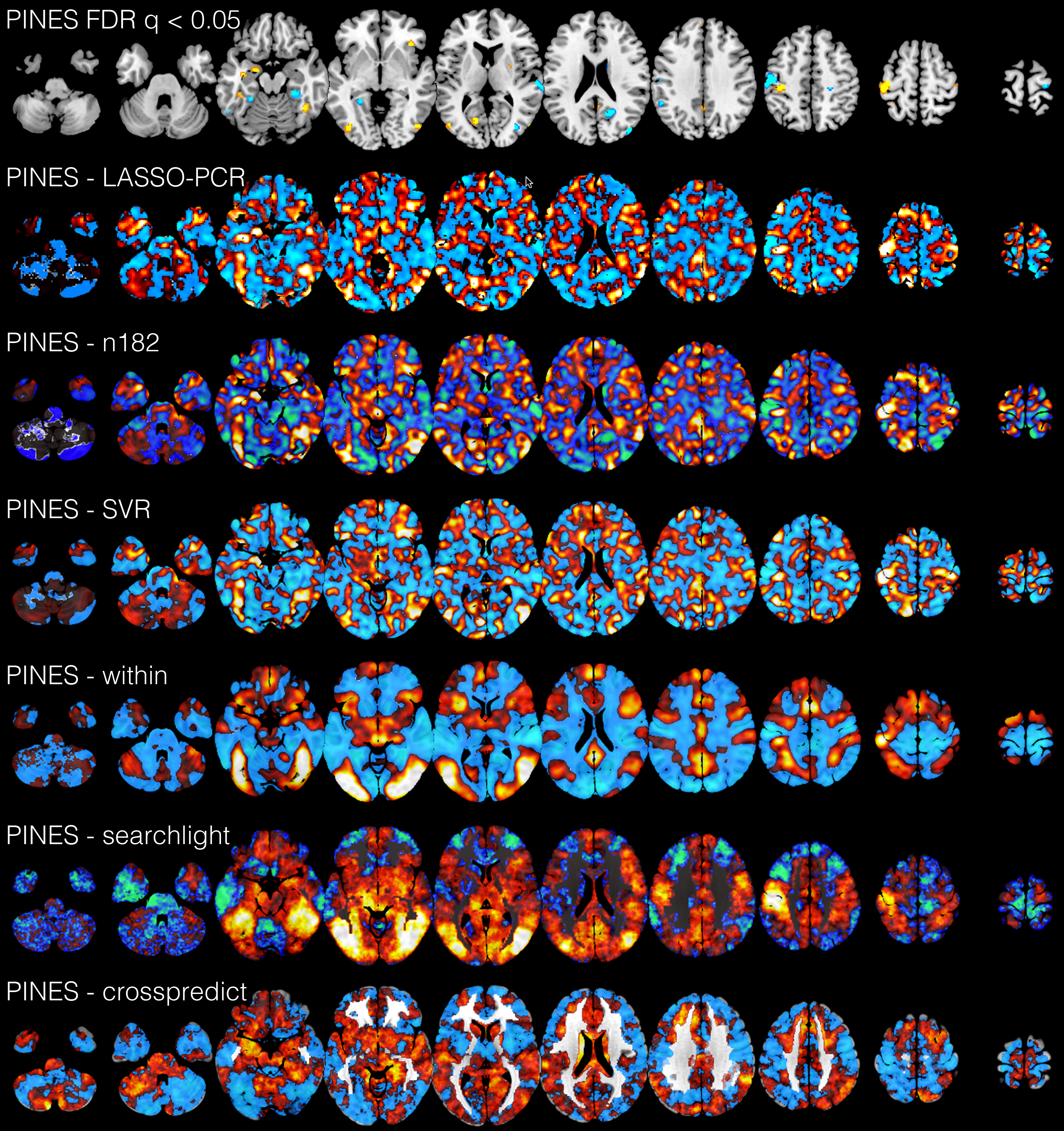

Supplement: S1 Fig — This figure depicts axial slice montages of different analytic techniques. PINES FDR: the PINES thresholded using a 5,000 sample bootstrap procedure at FDR q < 0.05 whole-brain corrected with a cluster extent k = 10. PINES-LASSO-PCR the full unthresholded PINES pattern trained with LASSO-PCR (n = 121). PINES-n182: the PINES weight map when it is trained with the full dataset (n = 182). PINES-SVR: the PINES when it is trained with the training data (n = 121) using support vector regression. PINES-within: the average weight map for the within-participant analysis, in which a separate pattern was trained for each participant to predict ratings to individual photos (n = 121). PINES-searchlight: standardized prediction values (i.e., correlations) for each voxel from a whole-brain searchlight analysis (n = 182). (TIF) [file pbio.1002180.s003.tif]

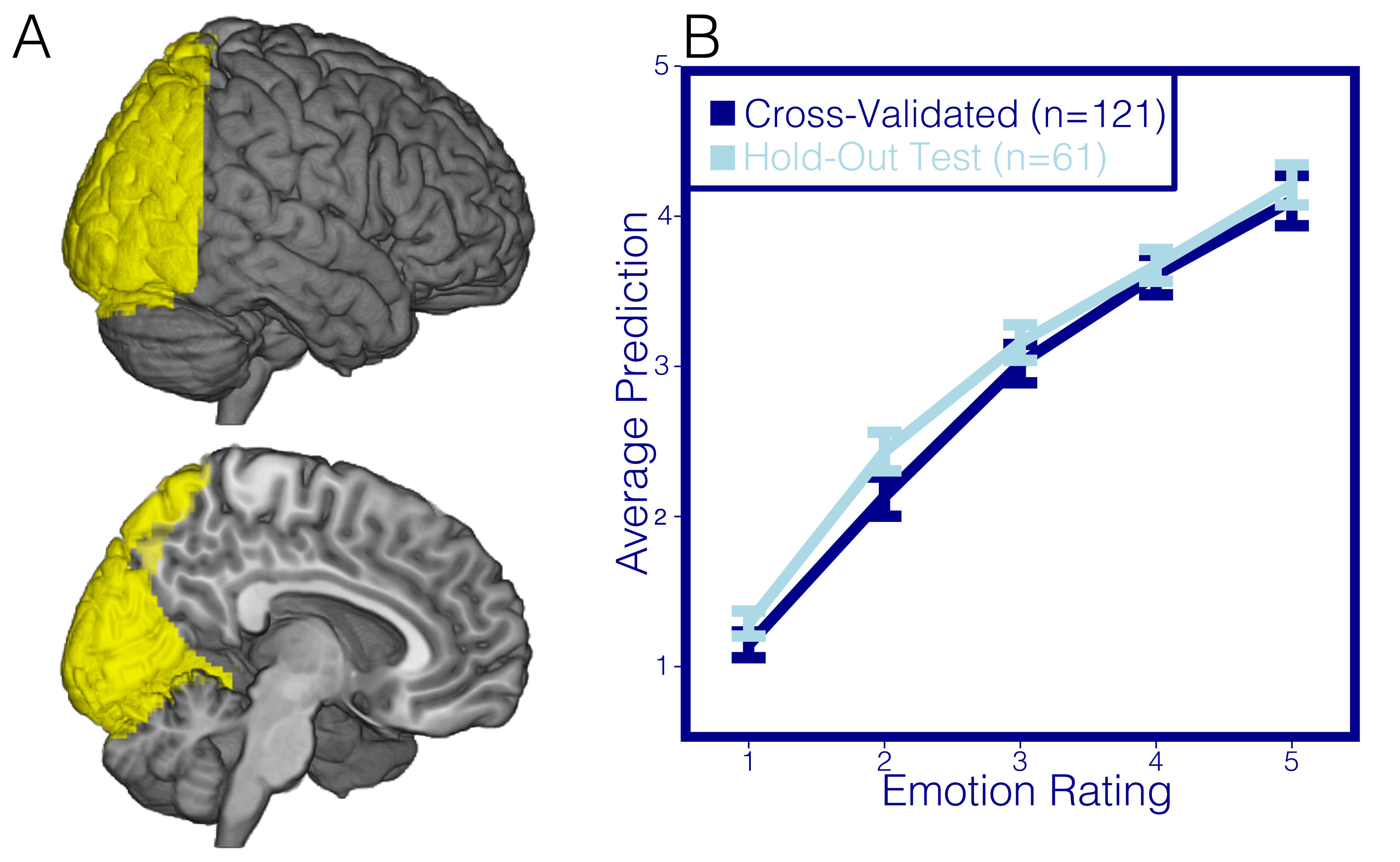

Supplement: S2 Fig — Panel A depicts the occipital mask excluded from the data prior to training the PINES. Panel B shows the predicted affective rating compared to the actual ratings for the cross validated participants (n = 121) and the separate holdout test dataset (n = 61). (TIF) [file pbio.1002180.s004.tif]

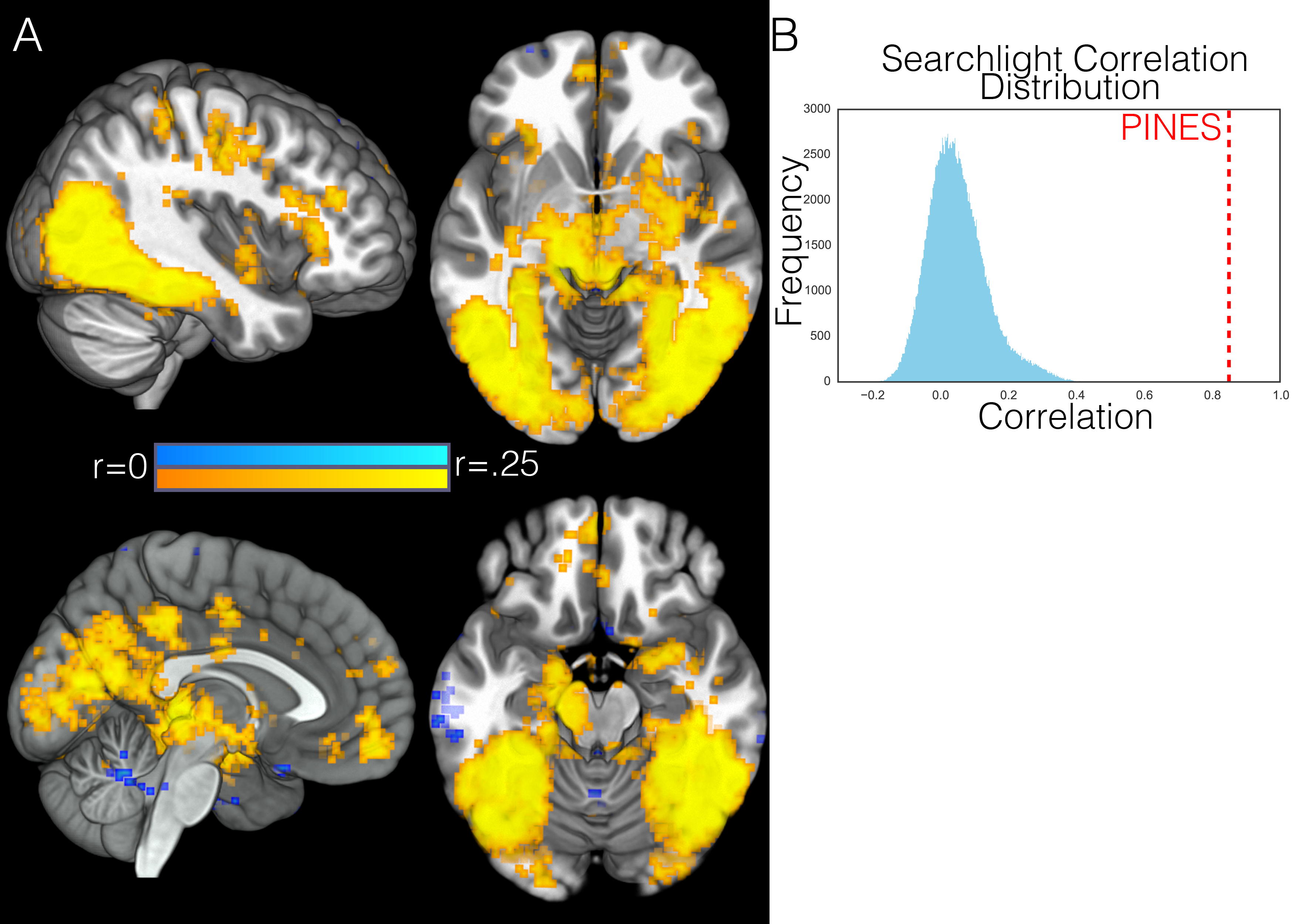

Supplement: S4 Fig — This figure depicts results from a whole-brain searchlight analysis in which we trained a searchlight (five voxel radius) to predict emotion rating using LASSO-PCR with the full dataset (n = 182) and 5-fold cross validation. Panel A shows the thresholded correlation values for each searchlight (p < 0.001, uncorrected). Panel B shows the distribution of the correlation values of all searchlights in the brain. The dotted line shows the cross validated PINES correlation (n = 121) for comparison. (TIF) [file pbio.1002180.s006.tif]

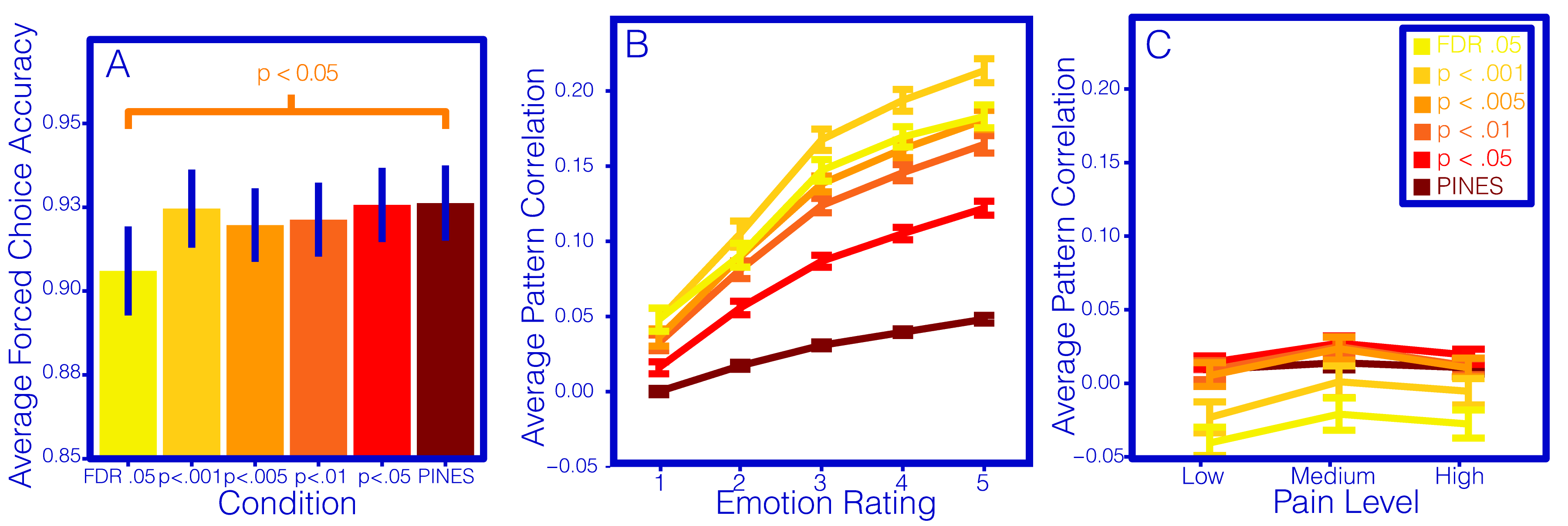

Supplement: S5 Fig — Panel A depicts the average forced-choice accuracy between high and low emotion ratings for the hold out test dataset (n = 61). The only threshold level that is significantly different from the PINES is the FDR q < 0.01 pattern. Panel B shows the average pattern correlation between each thresholded pattern for each emotion level. Panel C shows the average pattern correlation between each thresholded pattern for each level of pain. Error bars reflect ±1 standard error. (TIF) [file pbio.1002180.s007.tif]

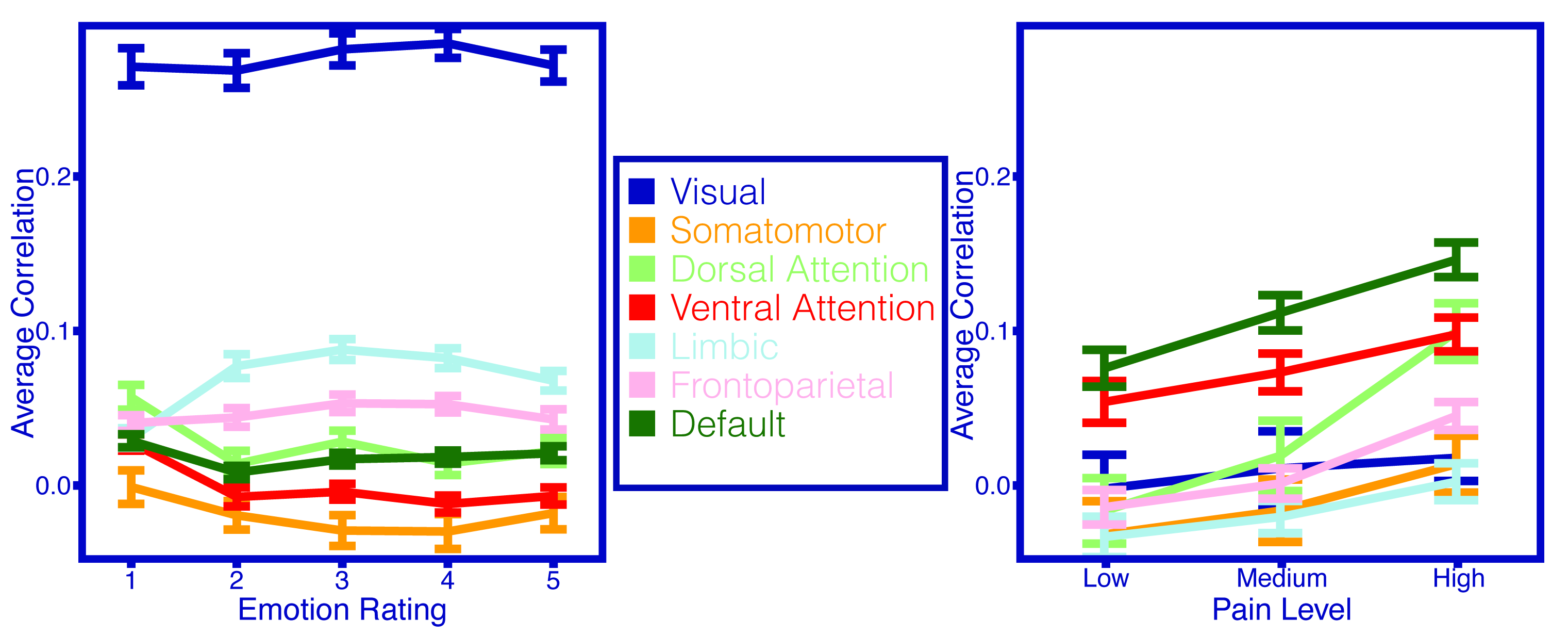

Supplement: S6 Fig — This figure examines how well resting-state networks perform using the same testing benchmarks as the PINES, NPS, and affective ROIs. The line plot depicts the pattern response of the network parcellation from Yeo et al., 2007 on emotion and pain test datasets using point-biserial spatial correlations. Panel A shows the average network predictions for each level of emotion, while panel B shows the average prediction for each level of pain. Error bars reflect ±1 standard error. (TIF) [file pbio.1002180.s008.tif]

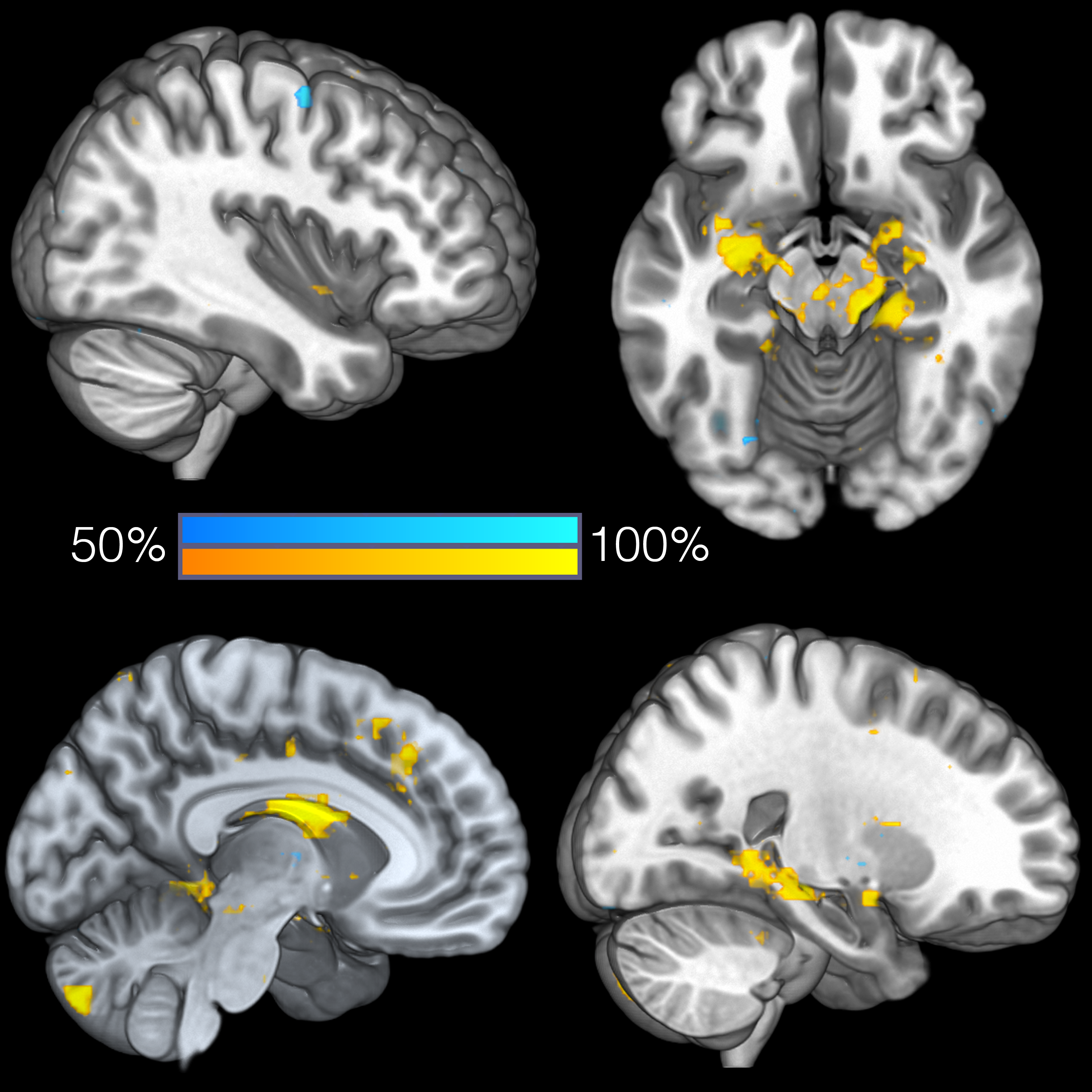

Supplement: S8 Fig — This figure depicts results from a whole-brain searchlight analysis in which we trained a searchlight (five voxel radius) to predict emotion rating using SVR with the training data set (n = 121) and 5-fold cross validation. We then applied each searchlight mask to the test pain data set (n = 28) to obtain a standardized pattern response, and calculated forced-choice accuracy within each participant to find searchlights that discriminated between high and low levels of pain. We show the accuracy results thresholded at p < 0.001 (note FDR q < 0.05 = p < 0.0015). (TIF) [file pbio.1002180.s010.tif]
