# Supplementary material for: A Sensitive and Specific Neural Signature for Picture-Induced Negative Affect
Source: PLoS Biol. 2015 Jun 22;13(6):e1002180. doi: 10.1371/journal.pbio.1002180 (PMC4476709; doi:10.1371/journal.pbio.1002180)
Supplement: S1 Methods — (DOCX) [file pbio.1002180.s011.docx]

**Supplemental Experimental Procedures and Results**

A sensitive and specific neural signature for picture-induced negative affect

Luke J. Chang, Peter J. Gianaros, Stephen B. Manuck, Anjali Krishnan, & Tor D. Wager

*PINES Comparison to Alternative Algorithms and Analysis Strategies*

Though we have previously demonstrated the validity of the LASSO-PCR algorithm in previous papers ([1](#_ENREF_1), [2](#_ENREF_2)), we ran several additional analyses to ensure that our results could not be explained by our algorithm selection or analytic strategy. First, we trained a support vector regression (SVR) algorithm using the same training and test procedure described in the methods (whole-brain between-subject prediction) and a previous publication validating the NPS ([2](#_ENREF_2)) and found that the weight map was virtually identical to the PINES with a spatial correlation of r=0.99. The SVR weight map also performed very well in terms of prediction. For the test sample, the average root mean squared error (RMSE) was 1.40±0.1 rating units (with standard error), and the average within-subject correlation between predicted and actual ratings was r = 0.92±0.01. The SVR weight map accurately classified highly aversive (rating 5) vs. non-aversive (rating 1) pictures with 100% forced-choice accuracy in the test sample, and 96% accuracy in discriminating between high emotion and high pain.

Second, to demonstrate that our cross-validation procedure did not bias our results, we ran an additional training with leave-one-subject out cross-validation with the entire data sample (n=182). This weight map was similar to the PINES with a spatial correlation of r=0.82 and also performed very well in terms of prediction. The average root mean squared error (RMSE) was 1.19±0.05 (standard error) rating units, and the average within-subject correlation between predicted and actual ratings was r = 0.89±0.01. The weight map accurately classified highly aversive (rating 5) vs. non-aversive (rating 1) pictures with 100% forced-choice accuracy in the cross-validation folds, and 89% accuracy in discriminating between high emotion and high pain.

Third, to assess the influence of the size of the training sample size on our results, we ran our LASSO-PCR prediction algorithm using a sample size of 2 – 121 participants. We ran each prediction 20 times using randomly sampled subjects (without replacement) and tested the performance of the pattern on the separate hold-out test sample (n=61). Fig. S3A illustrates the sensitivity of each pattern in predicting emotion rating. The plot depicts the average standardized prediction of emotion ratings with the error bars reflecting one standard deviation across the mean of the 20 samples. Fig. S3B illustrates the specificity of the pattern with respect to pain. We calculated the average single-interval accuracy of the pattern’s ability to discriminate between high and low levels of pain. Fig. S3C shows the average single-interval accuracy in discriminating between high levels of emotion and pain. Fig. S3D depicts the average spatial correlation of the pattern with the PINES. Overall, the prediction accuracy is remarkably robust to using small training samples in this particular dataset. First, the pattern is highly sensitive to detecting responses to emotion. The standardized prediction for emotion ratings increases quite rapidly and reaches r = 0.9 with 52 participants. This may be because the high vs low emotion data produces an extremely robust and consistent activation profile across individuals. We would not expect this to hold for all samples and tasks, and in many cases larger samples would likely yield substantially improved prediction results. Second, the pattern is highly specific with respect to pain. On average the pattern discriminates around 60% between high and low levels of pain, though there is considerably more variability when the pattern is trained using smaller sample sizes. The pattern was also able to discriminate between high levels of pain and emotion even when it was trained on very small sample sizes. Finally, as expected, the robustness of the spatial pattern was much more sensitive to the training sample size, and the weight map became more stable and consistent approximately linearly as a function of sample size.  This suggests that neuroscientific inferences on the locations of the regions that are most predictive will benefit substantially from using larger training samples.

*Searchlight Analysis*

We compared our between subject whole brain prediction approach with the popular searchlight approach ([3](#_ENREF_3)) to examine the performance of individual regions in the brain in predicting affective ratings. We used a whole brain searchlight approach (sphere with 5 voxel radius) to identify local patterns of activations that were predictive of emotion ratings. This entailed using LASSO-PCR with 5 fold cross-validation across the entire sample (n=182). In contrast to our whole brain analyses, we used a grey matter mask to reduce the search space. The full weight map can be seen in Fig. S1 and correlations thresholded at p < 0.001 uncorrected at each voxel can be seen in Fig. S4A. Interestingly, there was no searchlight that achieved a correlation near the prediction of our whole brain PINES model (Fig. S4B, correlation range [-0.22, 0.44]. Overall, the searchlight approach revealed largely similar regions as the PINES weights. However, the results occasionally diverged quite strongly even in terms of sign of the relationship. We believe that this is a consequence of the searchlight making voxel-wise and not distributed inferences. The weights from the PINES reflect independent contributions to the prediction controlling for all other voxels and removes shared variance among voxels. Searchlight voxels only reflect local patterns and do not consider processes occurring in more distant areas of the brain. These results provide additional support for the notion that no single anatomical region can solely account for the experience of emotion, rather this appears to recruit multiple systems distributed throughout the brain. We believe this is why the PINES better predicts emotion ratings than any given anatomical region: It incorporates information distributed across brain networks.

*Cross-prediction Searchlight Analysis*

An additional question is whether any local information predictive of emotional experience can cross-predict pain intensity experience. In this analysis, we used a whole brain searchlight approach (sphere with 5 voxel radius) using voxels within a grey matter mask and only the training sample (n=121). For each searchlight, we used Support Vector Regression (SVR) with 5-fold cross-validation to predict emotion ratings as it is considerably faster than LASSO-PCR and results in similar solutions (see above). We then applied each searchlight to the pain dataset to calculate a standardized pattern response and used forced choice accuracy to calculate how well each searchlight could distinguish between high and low levels of pain in the test dataset. The unthresholded results can be seen in Fig S1, while the thresholded results can be viewed in Fig S8. Overall, we find that local patterns predictive of emotion ratings in the amygdala, ACC, parahippocampal cortex, and caudate generalize to pain experience. These results are consistent with our anatomical ROI analyses as well as Woo et al. 2015 ([4](#_ENREF_4)), which suggests that parahippocampal/striatal systems may be encoding processes related to general negative affect/arousal. However, we believe these results should be interpreted cautiously for several reasons. First, as these regions by themselves were not highly predictive of emotion (e.g., dACC), it is unclear what processes they actually represent. We contend that they are likely related to processing salience or arousal ([5](#_ENREF_5), [6](#_ENREF_6)). Second, even though we found evidence of shared processing in these regions, it does not preclude the possibility of finding *non-shared* patterns in these regions as well. For example, cellular-level data suggests that there are likely both non-specific and specific neural codes in the same broad regions ([7](#_ENREF_7), [8](#_ENREF_8)).

*Thresholded Pattern Performance*

The PINES includes non-zero weights in voxels across the brain (Fig. S1), though it is primarily driven by high weights in specific regions (Fig. 2A). The *a priori* division of our data into cross-validation and test sets allows us to address whether a more sparse (and therefore interpretable) pattern might perform sufficiently well (e.g., [Grosenick, Greer (9](#_ENREF_9))). We thresholded the PINES using a 5000-sample bootstrap procedure at FDR corrected q < 0.05 and uncorrected p < 0.001, 0.005, 0.01, and 0.05 levels and tested the pattern expression on the independent emotion and pain test datasets (Fig. S5). The pattern’s overall accuracy drops as it becomes more sparse, suggesting that contributions from many voxels are important, but the pattern specifically predicts emotional experience (not pain) even when the pattern map is thresholded at p < 0.001 and only contains of 1.6% of the number of voxels of the full PINES (as shown in Fig. 2; see also Table 2). This finding suggests that the majority of the voxels are not making substantive contributions to the overall prediction, and a sparse representation is achievable. It is important to note that the specificity of the PINES slightly drops as the pattern becomes more sparse. Though there is a large effect between pattern responses to low and high emotion, and a low response to pain, the PINES pattern can still slightly discriminate between high and low levels of pain (see Table 2). Thus, while the thresholded patterns can be useful for interpreting the brain regions that most reliably make the strongest contribution to the prediction, it is generally preferable to use the full PINES pattern when making predictions when possible.

*Anatomical ROIs Show a Non-Monotonic Response Function to Emotion*

Fig. 4B reveals that the anatomical ROIs may have a non-monotonic response to levels of emotion. To test this, we used a random intercept mixed effects polynomial regression model to predict average ROI activity from a linear combination of subjective ratings (centered) and their quadratic. Indeed, the amygdala and insula activity was significantly explained by a linear combination of both ratings, $\hat{\beta}$ = 0.03±0.01, t(213) = 3.31, p = 0.001, and squared ratings, $\hat{\beta}$ = -0.02±0.01, t(213) = -2.55, p = 0.01 (amygdala); ratings, $\hat{\beta}$ = 0.04±0.01, t(212) = 5.00, p < 0.001, and squared ratings, $\hat{\beta}$ = -0.02±0.01, t(216) = -3.23, p = 0.001 (insula). Responses in the ACC, however, were more monotonic as ratings significantly explained activation, $\hat{\beta}$ = 0.03±0.01, t(213) = 3.24, p = 0.001, but not squared ratings, $\hat{\beta}$ = -0.00±0.00, t(218) = -0.02, p = 0.98. This suggests that the amygdala and insula, but not the ACC have an inverse u-shaped response to emotion.

**Supplemental References**

1. Wager TD, Atlas LY, Leotti LA, Rilling JK. Predicting individual differences in placebo analgesia: contributions of brain activity during anticipation and pain experience. The Journal of neuroscience : the official journal of the Society for Neuroscience. 2011;31(2):439-52. Epub 2011/01/14. doi: 10.1523/JNEUROSCI.3420-10.2011. PubMed PMID: 21228154.

2. Wager TD, Atlas LY, Lindquist MA, Roy M, Woo CW, Kross E. An fMRI-based neurologic signature of physical pain. The New England journal of medicine. 2013;368(15):1388-97. doi: 10.1056/NEJMoa1204471. PubMed PMID: 23574118.

3. Kriegeskorte N, Goebel R, Bandettini P. Information-based functional brain mapping. Proc Natl Acad Sci U S A. 2006;103(10):3863-8. doi: 10.1073/pnas.0600244103. PubMed PMID: 16537458; PubMed Central PMCID: PMC1383651.

4. Woo CW, Koban L, Kross E, Lindquist MA, Banich MT, Ruzic L, et al. Separate neural representations for physical pain and social rejection. Nature communications. 2014;5:5380. doi: 10.1038/ncomms6380. PubMed PMID: 25400102; PubMed Central PMCID: PMC4285151.

5. Seeley WW, Menon V, Schatzberg AF, Keller J, Glover GH, Kenna H, et al. Dissociable intrinsic connectivity networks for salience processing and executive control. Journal of Neuroscience. 2007;27(9):2349-56. Epub 2007/03/03. doi: 27/9/2349 [pii]

10.1523/JNEUROSCI.5587-06.2007. PubMed PMID: 17329432; PubMed Central PMCID: PMC2680293.

6. Menon V, Uddin LQ. Saliency, switching, attention and control: a network model of insula function. Brain structure & function. 2010;214(5-6):655-67. Epub 2010/06/01. doi: 10.1007/s00429-010-0262-0. PubMed PMID: 20512370; PubMed Central PMCID: PMC2899886.

7. Kvitsiani D, Ranade S, Hangya B, Taniguchi H, Huang JZ, Kepecs A. Distinct behavioural and network correlates of two interneuron types in prefrontal cortex. Nature. 2013;498(7454):363-+. doi: Doi 10.1038/Nature12176. PubMed PMID: WOS:000320592900040.

8. Sikes RW, Vogt BA. Nociceptive Neurons in Area-24 of Rabbit Cingulate Cortex. Journal of neurophysiology. 1992;68(5):1720-32. PubMed PMID: WOS:A1992JY68500019.

9. Grosenick L, Greer S, Knutson B. Interpretable classifiers for FMRI improve prediction of purchases. IEEE transactions on neural systems and rehabilitation engineering : a publication of the IEEE Engineering in Medicine and Biology Society. 2008;16(6):539-48. doi: 10.1109/TNSRE.2008.926701. PubMed PMID: 19144586.
