# Supplementary material for: A Sensitive and Specific Neural Signature for Picture-Induced Negative Affect
Source: PLoS Biol. 2015 Jun 22;13(6):e1002180. doi: 10.1371/journal.pbio.1002180 (PMC4476709; doi:10.1371/journal.pbio.1002180)
Supplement: S1 Table — All balanced accuracies reported in this table result from forced-choice classification on the test dataset (n = 47). This analysis excludes participants that did not make a rating of either “1” or “5.” +indicates that accuracy is significantly different from chance (50%) using a two-tailed independent samples binomial test. *indicates accuracy significantly different from PINES performance using a two-sample, two-tailed z-test for proportions. (DOCX) [file pbio.1002180.s012.docx]

Table S1. Pattern Forced Choice Classification

|  | Map | Emotion - 5 v 1 (SE) | Emotion - 5 v 3 (SE) | Emotion - 3 v 1 (SE) |
| --- | --- | --- | --- | --- |
| Pattern |  |  |  |  |
|  | PINES | 100 (0%)+ | 90.7 (4.4%)+ | 100 (0%)+ |
|  | NPS | 19.1 (5.7%)+* | 69.8 (7%)+* | 8.8 (3.7%)+* |
| Average ROI |  |  |  |  |
|  | Amygdala | 70.2 (6.7%)+* | 44.2 (7.6%)* | 75.4 (5.7%)+* |
|  | ACC | 63.8 (7%)* | 53.5 (7.6%)* | 71.9 (6%)+* |
|  | Insula | 68.1 (6.8%)+* | 53.5 (7.6%)* | 70.2 (6.1%)+* |
| Network |  |  |  |  |
|  | Visual | 44.7 (7.3%)* | 55.8 (7.6%)* | 49.1 (6.6%)* |
|  | Somatomotor | 17 (5.5%)+* | 60.5 (7.5%)* | 17.5 (5%)+* |
|  | Dorsal Attention | 63.8 (7%)* | 27.9 (6.8%)+* | 93 (3.4%)+ |
|  | Ventral Attention (Salience) | 57.4 (7.2%)* | 55.8 (7.6%)* | 52.6 (6.6%)* |
|  | Limbic | 72.3 (6.5%)+* | 60.5 (7.5%)* | 70.2 (6.1%)+* |
|  | Frontoparietal | 61.7 (7.1%)* | 41.9 (7.5%)* | 82.5 (5%)+* |
|  | Default | 78.7 (6%)+* | 41.9 (7.5%)* | 86 (4.6%)+* |
