# Supplementary material for: A Sensitive and Specific Neural Signature for Picture-Induced Negative Affect
Source: PLoS Biol. 2015 Jun 22;13(6):e1002180. doi: 10.1371/journal.pbio.1002180 (PMC4476709; doi:10.1371/journal.pbio.1002180)
Supplement: S2 Table — All balanced accuracies reported in this table result from forced-choice classification on the test dataset (n = 47). This analysis excludes participants that did not make a rating of either “1” or “5.” +indicates that accuracy is significantly different from chance (50%) using a two-tailed independent samples binomial test. *indicates accuracy significantly different from PINES performance using a two-sample, two-tailed z-test for proportions. (DOCX) [file pbio.1002180.s013.docx]

Table S2. Virtual Lesion Forced Choice Classification

|  | Map | nVoxels | Emotion - 5 v 1 (SE) | Emotion - 5 v 3 (SE) | Emotion - 3 v 1 (SE) |
| --- | --- | --- | --- | --- | --- |
| Pattern |  |  |  |  |  |
|  | PINES | 328796 | 100 (0%)+ | 90.7 (4.4%)+ | 100 (0%)+ |
|  | PINES (p < .001) | 5303 | 100 (0%)+ | 90.7 (4.4%)+ | 100 (0%)+ |
| Single Cluster |  |  |  |  |  |
|  | Visual (LOC) | 981 | 97.9 (2.1%)+ | 62.8 (7.4%)* | 98.2 (1.7%)+ |
|  | Somatosensory/STG | 308 | 68.1 (6.8%)+* | 53.5 (7.6%)* | 63.2 (6.4%)* |
|  | Sensorimotor/V1 | 335 | 61.7 (7.1%)* | 41.9 (7.5%)* | 66.7 (6.2%)+* |
|  | DMPFC/PCC | 318 | 76.6 (6.2%)+* | 55.8 (7.6%)* | 87.7 (4.3%)+* |
|  | Sensorimotor/Cerebellum | 1227 | 95.7 (2.9%)+ | 79.1 (6.2%)+ | 89.5 (4.1%)+* |
|  | Parahippocampal Gyrus | 1025 | 46.8 (7.3%)* | 76.7 (6.4%)+ | 28.1 (6%)+* |
|  | Occipital Pole | 118 | 63.8 (7%)* | 51.2 (7.6%)* | 75.4 (5.7%)+* |
|  | Precuneus/Caudate | 537 | 46.8 (7.3%)* | 72.1 (6.8%)+ | 19.3 (5.2%)+* |
|  | Amygdala/Insula | 454 | 72.3 (6.5%)+* | 46.5 (7.6%)* | 80.7 (5.2%)+* |
|  |  |  |  |  |  |
| Virtual Lesion | Visual (LOC) | 4322 | 95.7 (2.9%)+ | 88.4 (4.9%)+ | 77.2 (5.6%)+* |
|  | Somatosensory/STG | 4995 | 100 (0%)+ | 86 (5.3%)+ | 100 (0%)+ |
|  | Sensorimotor/V1 | 4968 | 100 (0%)+ | 93 (3.9%)+ | 100 (0%)+ |
|  | DMPFC/PCC | 4985 | 100 (0%)+ | 86 (5.3%)+ | 100 (0%)+ |
|  | Sensorimotor/Cerebellum | 4076 | 100 (0%)+ | 81.4 (5.9%)+ | 100 (0%)+ |
|  | Parahippocampal Gyrus | 4278 | 100 (0%)+ | 79.1 (6.2%)+ | 100 (0%)+ |
|  | Occipital Pole | 5185 | 100 (0%)+ | 90.7 (4.4%)+ | 100 (0%)+ |
|  | Precuneus/Caudate | 4766 | 100(0%)+ | 79.1(6.2%)+ | 100(0%)+ |
|  | Amygdala/Insula | 4849 | 100(0%)+ | 93(3.9%)+ | 100(0%)+ |
