# Supplementary material for: A Sensitive and Specific Neural Signature for Picture-Induced Negative Affect
Source: PLoS Biol. 2015 Jun 22;13(6):e1002180. doi: 10.1371/journal.pbio.1002180 (PMC4476709; doi:10.1371/journal.pbio.1002180)
Supplement: S3 Table — (DOCX) [file pbio.1002180.s014.docx]

Table S3. Thresholded PINES Clusters (p < 0.001, k=10)

| Name | Cluster | Cluster Number | Hemisphere | X | Y | Z | nVoxels | Volume mm^3^ | Max Weight |
| --- | --- | --- | --- | --- | --- | --- | --- | --- | --- |
| Cerebellum - V | Sensorimotor | 5 | R | 20 | -48 | -24 | 83 | 664 | 4.86 |
| Inferior Lateral Occipital Cortex | Lateral Occipital Cortex | 1 | L | -42 | -78 | -4 | 505 | 4040 | 4.88 |
| Inferior Temporal Gyrus | Precuneus, Caudate | 8 | L | -56 | -56 | -20 | 90 | 720 | -4.03 |
| Temporal Fusiform Gyrus/Parahippocampal Gyrus | Parahippocampal Gyrus | 6 | L | -26 | -40 | -16 | 225 | 1800 | -4.53 |
| Parahippocampal Gyrus | Parahippocampal Gyrus | 6 | R | 28 | -38 | -14 | 403 | 3224 | -4.77 |
| Amygdala | Amygdala, Insula | 9 | L | -18 | -4 | -18 | 159 | 1272 | 5.39 |
| Temporal Occipital Fusiform | Lateral Occipital Cortex | 1 | R | 42 | -52 | -16 | 156 | 1248 | 4.44 |
| Anterior Hippocampus/White Matter | Amygdala, Insula | 9 | L | -36 | -10 | -14 | 99 | 792 | 4.66 |
| Inferior Lateral Occipital Cortex | Lateral Occipital Cortex | 1 | R | 52 | -72 | -2 | 320 | 2560 | 4.39 |
| Anterior Insula/Lateral Orbitofrontal Cortex | Amygdala, Insula | 9 | R | 38 | 28 | -4 | 111 | 888 | 4.07 |
| Superior Temporal Gyrus | Somatosensory/STG | 2 | R | 66 | -24 | 8 | 219 | 1752 | -4.8 |
| V1, Intracalcarine Sulcus | Sensorimotor, V1 | 3 | L | -16 | -68 | 6 | 77 | 616 | 4.56 |
| Occipital Pole | Occipital Pole | 7 | L | -34 | -96 | 10 | 59 | 472 | 3.91 |
| Occipital Pole | Parahippocampal Gyrus | 6 | R | 38 | -78 | 16 | 266 | 2128 | -4.76 |
| Putamen | Amygdala, Insula | 9 | R | 26 | 0 | 12 | 85 | 680 | 4.29 |
| SII | Sensorimotor, V1 | 3 | L | -56 | -20 | 14 | 44 | 352 | 4.2 |
| Occipital Pole | Occipital Pole | 7 | R | 22 | -94 | 16 | 59 | 472 | 5.07 |
| Precuneous | Precuneus, Caudate | 8 | R | 18 | -58 | 20 | 183 | 1464 | -5.4 |
| Posterior Cingulate Cortex | DMPFC, PCC | 4 | R/L | 0 | -50 | 28 | 170 | 1360 | 4.5 |
| Caudate | Precuneus, Caudate | 8 | R | 12 | 0 | 18 | 89 | 712 | -4.58 |
| Superior Lateral Occipital Cortex | Parahippocampal Gyrus | 6 | L | -36 | -90 | 26 | 131 | 1048 | -4.24 |
| Dorsomedial PFC/Superior Frontal Gyrus | DMPFC, PCC | 4 | L | -6 | 54 | 28 | 75 | 600 | 4.25 |
| Temporal Parietal Junction/Supramarginal Gyrus | Precuneus, Caudate | 8 | L | -54 | -46 | 34 | 73 | 584 | -4.39 |
| Postcentral Gyrus | Sensorimotor | 5 | L | -46 | -22 | 52 | 1057 | 8456 | 7.92 |
| Superior Lateral Occipital Cortex | Precuneus, Caudate | 8 | R | 14 | -66 | 54 | 102 | 816 | -4.51 |
| Superior Parietal Lobule | Sensorimotor, V1 | 3 | L | -30 | -44 | 56 | 107 | 856 | 5.55 |
| Postcentral Gyrus | Sensorimotor, V1 | 3 | R | 42 | -24 | 56 | 107 | 856 | 4.63 |
| Superior Frontal Gyrus | DMPFC, PCC | 4 | L | -6 | 16 | 56 | 73 | 584 | 4.05 |
| Precentral Gyrus | Sensorimotor | 5 | L | -30 | -6 | 70 | 87 | 696 | 4.24 |
| Precentral Gyrus | Somatosensory/STG | 2 | R | 22 | -26 | 74 | 89 | 712 | -4.41 |
